# Spirometric pattern and cardiovascular risk: a prospective study of 0.3 million Chinese never-smokers

**DOI:** 10.1016/j.lanwpc.2024.101274

**Published:** 2024-12-30

**Authors:** Yinqi Ding, Jingcen Hu, Canqing Yu, Dianjianyi Sun, Pei Pei, Ling Yang, Yiping Chen, Huaidong Du, Libo Zhang, Dan Schmidt, Maxim Barnard, Junshi Chen, Zhengming Chen, Liming Li, Jun Lv, China Kadoorie Biobank Collaborative Group, Junshi Chen, Zhengming Chen, Robert Clarke, Rory Collins, Liming Li, Jun Lv, Richard Peto, Robin Walters, Daniel Avery, Maxim Barnard, Derrick Bennett, Ruth Boxall, Ka Hung Chan, Yiping Chen, Zhengming Chen, Charlotte Clarke, Johnathan Clarke, Robert Clarke, Huaidong Du, Ahmed Edris Mohamed, Hannah Fry, Simon Gilbert, Pek Kei Im, Andri Iona, Maria Kakkoura, Christiana Kartsonaki, Kshitij Kolhe, Hubert Lam, Kuang Lin, James Liu, Mohsen Mazidi, Iona Millwood, Sam Morris, Qunhua Nie, Alfred Pozarickij, Maryam Rahmati, Paul Ryder, Dan Schmidt, Becky Stevens, Iain Turnbull, Robin Walters, Baihan Wang, Lin Wang, Neil Wright, Ling Yang, Xiaoming Yang, Pang Yao, Xiao Han, Can Hou, Qingmei Xia, Chao Liu, Jun Lv, Pei Pei, Dianjianyi Sun, Canqing Yu, Lang Pan, Zengchang Pang, Ruqin Gao, Shanpeng Li, Haiping Duan, Shaojie Wang, Yongmei Liu, Ranran Du, Yajing Zang, Liang Cheng, Xiaocao Tian, Hua Zhang, Yaoming Zhai, Feng Ning, Xiaohui Sun, Feifei Li, Silu Lv, Junzheng Wang, Wei Hou, Wei Sun, Shichun Yan, Xiaoming Cui, Chi Wang, Zhenyuan Wu, Yanjie Li, Quan Kang, Huiming Luo, Tingting Ou, Xiangyang Zheng, Zhendong Guo, Shukuan Wu, Yilei Li, Huimei Li, Ming Wu, Yonglin Zhou, Jinyi Zhou, Ran Tao, Jie Yang, Jian Su, Fang Liu, Jun Zhang, Yihe Hu, Yan Lu, Liangcai Ma, Aiyu Tang, Shuo Zhang, Jianrong Jin, Jingchao Liu, Mei Lin, Zhenzhen Lu, Lifang Zhou, Changping Xie, Jian Lan, Tingping Zhu, Yun Liu, Liuping Wei, Liyuan Zhou, Ningyu Chen, Yulu Qin, Sisi Wang, Xianping Wu, Ningmei Zhang, Xiaofang Chen, Xiaoyu Chang, Mingqiang Yuan, Xia Wu, Xiaofang Chen, Wei Jiang, Jiaqiu Liu, Qiang Sun, Faqing Chen, Xiaolan Ren, Caixia Dong, Hui Zhang, Enke Mao, Xiaoping Wang, Tao Wang, Xi Zhang, Kai Kang, Shixian Feng, Huizi Tian, Lei Fan, XiaoLin Li, Huarong Sun, Pan He, Xukui Zhang, Min Yu, Ruying Hu, Hao Wang, Xiaoyi Zhang, Yuan Cao, Kaixu Xie, Lingli Chen, Dun Shen, Xiaojun Li, Donghui Jin, Li Yin, Huilin Liu, Zhongxi Fu, Xin Xu, Hao Zhang, Jianwei Chen, Yuan Peng, Libo Zhang, Chan Qu, Shuya Li, Haiqiang Qin, Yongjun Wang, Qiling Chen, Jihua Wang, Xiaojia Sun, Lei Wang, Xun Wang, Liming Zhang, Shanshan Zhou, Hongyuan Chen, Li Chen, Haiyan Gou, Weizhi Wang, Yanmei Zhu, Yulan Zhu, Ning Zhang, Xin Cheng, Qiang Dong, Yi Dong, Kun Fang, Yiting Mao, Yu An, Peiling Chen, Yinghua Chen, Zhihong Liu, Lihua Zhang, Xiaohong Chen, Naixin Jv, Xiaojiu Li, Liyang Liu, Yun Lu, Xiaona Xing, Shihao You, Xiaoli Cheng, Chaojun Gua, Jinping Jiang, Jingyi Liu, Shumei Ma, Xuefeng Yang, Xiaomo Du, Jian Xu, Xuecheng Yang, Xiaodi Zhao, Zilong Hao, Ming Liu, Deren Wang, Xiaoting Li, Lili Hui, Zhanling Liao, Feng Liu, Chunning Feng, Dejiang Ji, Fengxia Qu, Wenwen Yuan, Xin Fu, Jing Ding, Peng Du, Lirong Jin, Yueshi Mao, Xin Wang

**Affiliations:** a Department of Epidemiology & Biostatistics, School of Public Health, Peking University, Beijing 100191, China; b Peking University Center for Public Health and Epidemic Preparedness & Response, Beijing 100191, China; c Key Laboratory of Epidemiology of Major Diseases (Peking University), Ministry of Education, China; d Clinical Trial Service Unit & Epidemiological Studies Unit (CTSU), Nuffield Department of Population Health, University of Oxford, United Kingdom; e Non-communicable Chronic Diseases Prevention and Control Department, Liuyang Center for Disease Control and Prevention, Hunan, 410300, China; f China National Center for Food Safety Risk Assessment, Beijing, China; g State Key Laboratory of Vascular Homeostasis and Remodeling, Peking University, China

**Keywords:** Chronic obstructive pulmonary disease, Restrictive lung function, Forced expiratory volume in one second, Cardiovascular disease, Obesity, Underweight

## Abstract

**Background:**

Existing studies have not provided robust evidence about the CVD risk of non-smoking patients with restrictive spirometric pattern (RSP) or airflow obstruction (AFO), and how the risk is modified by body shape. We aimed to bridge the gap.

**Methods:**

We used never-smokers' data from the China Kadoorie Biobank (CKB) and performed Cox models by sex (278,953 females and 50,845 males). Spirometry was used to assess the baseline spirometric pattern. CVD outcomes were captured through multiple sources.

**Findings:**

Females’ results were presented primarily, though males' results were similar. During a median 12-year (maximum 14.5 years) follow-up, both RSP and AFO patients had increased risks of acute myocardial infarction (AMI), other ischaemic heart disease (other IHD), heart failure, pulmonary heart disease, arrhythmia, and intracerebral haemorrhage (ICH). RSP was also associated with ischaemic stroke (IS). The HRs (95% CIs) for AFO in females ranged from 1.29 (1.15–1.45) for ICH to 8.84 (7.79–10.03) for pulmonary heart disease, while those for RSP ranged from 1.11 (1.08–1.15) for IS to 3.17 (2.80–3.59) for pulmonary heart disease. These risks increased with the severity of AFO and reduced FVC. RSP/AFO was more strongly associated with other IHD, heart failure, and pulmonary heart disease in underweight females than in normal and obese counterparts, respectively.

**Interpretation:**

With the confounding of smoking fully controlled, both RSP and AFO were associated with higher risks of various CVD outcomes, which further increased with the severity of AFO and reduced FVC. These associations were even stronger in underweight individuals.

**Funding:**

National Natural Science Foundation of China, 10.13039/501100012166National Key Research and Development Program of China, Ministry of Science and Technology of the People's Republic of China, 10.13039/501100017647Kadoorie Charitable Foundation, UK Wellcome Trust, 10.13039/501100000265UK Medical Research Council, 10.13039/501100000289Cancer Research UK, and 10.13039/501100000274British Heart Foundation.

**Research in context**

**Evidence before this study**

We searched PubMed for existing articles published from inception to January 19, 2024, using spirometric pattern-related terms (“COPD” OR “airflow obstruction” OR “restrictive spirometric pattern”) and outcome terms (“cardiovascular disease” OR “heart disease” OR “heart failure” OR “cerebrovascular disease” OR “stroke” OR “arrhythmia”) combined with (“never smoker” OR “non-smoker”). The study type and language were not restricted. We also checked the reference lists of the retrieved articles to supplement the search results. Only four studies conducted analysis on COPD and CVD in never-smokers. Except for one study that found a negative relationship between FEV1/FVC quartiles and subarachnoid haemorrhage, the other studies found no associations of COPD with ischaemic stroke, ischaemic heart disease, myocardial infarction, and heart failure, presumably due to the small number of cases in never-smokers. Three prospective studies discovered positive associations of RSP with heart failure and composite CVD, however, the confounding of smoking was not adequately addressed. Overall, the CVD risk of nonsmoking patients with aberrant spirometry, particularly RSP, remains unknown.

**Added value of this study**

In this large cohort study of only never-smokers, we found that both RSP and AFO patients had elevated risks of various CVD outcomes, which increased with the severity of AFO and reduced FVC. Underweight females had stronger associations of RSP/AFO with ischaemic heart disease, heart failure, and pulmonary heart disease compared to their normal and obese counterparts. To the best of our knowledge, this is the first and largest study to examine the associations between spirometric patterns and a range of CVD outcomes in never-smokers. The restriction of participants removed the influence of smoking, and comprehensive analyses across all severity grades and various subgroups provided more robust evidence supporting the presence of a relationship and additional in-depth insights.

**Implications of all the available evidence**

Even in never-smokers, abnormal spirometric patterns raised CVD risk, especially among the underweight. The findings suggest the need for effective clinical approaches, alongside tobacco cessation, to enhance the prevention and management of cardiovascular risk in patients with pulmonary diseases. Furthermore, particular attention should be paid to underweight patients with lung disease.

## Introduction

Pulmonary diseases—both obstructive and restrictive—frequently coexist with cardiovascular disease (CVD).^1^ The burden of these two diseases in China is among the highest in the world.^2^^,^^3^ The coexistence of the two diseases complicates patients’ therapy and worsens their quality of life and prognosis compared to either condition existing alone.^4^ To reduce the risk of poor outcomes, it is critical to avoid the transition from an isolated condition to such multimorbidity.

Airflow obstruction (AFO) is a key feature of chronic obstructive pulmonary disease (COPD). The association between COPD and CVD has been investigated, but it is unclear whether COPD directly increases the likelihood of developing CVD through a mechanism other than smoking.^4^ Furthermore, approximately half of all COPD patients worldwide do not smoke,^5^ and their risk of CVD is unknown. Only four studies on the associations between COPD and CVD either used only never-smokers as study participants^6^ or performed stratified analysis by smoking,^7^, ^8^, ^9^ and the majority of them failed to find statistically significant associations, presumably due to small sample sizes.^6^, ^7^, ^8^ Changes in the lung parenchyma, pleura, chest wall, or neuromuscular apparatus can cause a restrictive spirometric pattern (RSP), which is typically defined as a decrease in lung volume and total lung capacity without evidence of airway obstruction.^1^ Only a few cohort studies have examined the association between RSP and CVD, and they may not have fully controlled for smoking-related confounding.^10^, ^11^, ^12^

Obesity is a crucial health risk factor in the general population, affecting several organ systems, and its link to cardiovascular disease morbidity and mortality is well established. However, observational studies in COPD patients found that low body mass index (BMI) was associated with a poor prognosis, whereas obesity was the opposite.^13^^,^^14^ This finding is known as the “obesity paradox in COPD”, in which smoking may cause bias.^15^^,^^16^ Thus, it is intriguing to investigate how body shape influences the association between pulmonary diseases and CVD, especially when biases introduced by including smokers were removed.

To completely control for the confounding effect of smoking, the current study restricted the analysis to never-smokers from the China Kadoorie Biobank (CKB). We aimed to examine the associations between abnormal spirometric patterns and the risks of various CVD outcomes, as well as how body shape modifies the effect of spirometric patterns on CVD risks.

## Methods

### Study design and participants

The study design and survey procedures of the CKB have been previously reported.^17^ In short, CKB is a population-based prospective cohort study that recruited 512,723 participants aged 30–79 from five urban and five rural regions across China. During the 2004–08 baseline survey, information was collected using an electronic interviewer-administered questionnaire that had built-in quality-control functions to minimize missing data and logical errors. Anthropometric measurements were taken by trained staff using standardized and regularly calibrated equipment. Following the completion of the baseline survey, a long-term follow-up was started for all participants. All the participants provided written informed consent before data collection. The study was approved by the Ethics Committee of the Chinese Center for Disease Control and Prevention (Beijing, China, number: 005/2004), the Peking University Health Science Center (Beijing, China, number: IRB00001052–20040), and the Oxford Tropical Research Ethics Committee of the University of Oxford (Oxford, UK, number: 025–04).

### Definition of spirometric patterns

At baseline, trained staff measured the participants’ pre-bronchodilator forced expiratory volume in one second (FEV1) and forced vital capacity (FVC) using a handheld Micro Spirometer in accordance with the recommended procedures.^18^^,^^19^ Two qualified measurements were recorded for each participant, with the highest value being used for analysis. A quality-control survey of 15,728 randomly selected participants was conducted 1–2 weeks after baseline, and the reproducibility of both FEV1 (*r*_*Spearman*_ = 0.913) and FVC (*r*_*Spearman*_ = 0.912) between the two surveys was satisfactory. Participants were also asked whether they had any of the following respiratory symptoms: shortness of breath while walking, slowing down while walking due to chest discomfort, coughing frequently last year, and coughing up sputum frequently last year.

To determine the optimal lung function reference equation for participants, we selected 43,783 healthy non-smokers (36,636 females and 7147 males) using the criteria described in the Supplementary methods. Spirometry z-scores and predicted values were calculated for each participant using the Global Lung Function Initiative (GLI) 2012 equations for South East Asian (SEA) and North East Asian (NEA),^20^ as well as the Chinese reference values.^21^ Despite a slight difference in lung function between our southern and northern populations, the SEA equation performed best in both populations (Supplementary Figs. S1 and S2 and Table S1), consistent with prior research findings.^22^ Therefore, the SEA equation was applied to all of the study participants.

Spirometric pattern was classified as: normal (FEV1/FVC ≥ 0.7 and FVC ≥ 80% predicted), with RSP (FEV1/FVC ≥ 0.7 and FVC < 80% predicted), and with AFO (FEV1/FVC < 0.7) (Table 1). As specified by the Global Initiative for Chronic Obstructive Lung Disease (GOLD),^23^ the severity of AFO was further classified into four grades based on FEV1 percent predicted (FEV1%P): GOLD 1 (FEV1%P ≥ 80%), GOLD 2 (50% ≤ FEV1%P < 80%), GOLD 3 (30% ≤ FEV1%P < 50%), and GOLD 4 (FEV1%P < 30%). The severity of reduced FVC was defined according to the 2022 American Thoracic Society/European Respiratory Society (ATS/ERS) technical standards^24^: normal (z-score > −1.65), mild (−2.5 ≤ z-score ≤ −1.65), moderate (−4 ≤ z-score < −2.5), and severe (z-score < −4).

**Table 1:** Categories and definitions of spirometric patterns and severity of reduced FVC and AFO.

Category	Definition
Spirometric patterns	
Normal	FEV1/FVC ≥ 0.7 and FVC ≥ 80% predicted
With RSP	FEV1/FVC ≥ 0.7 and FVC < 80% predicted
With AFO	FEV1/FVC < 0.7
Severity of reduced FVC	
Normal	FEV1/FVC ≥ 0.7 and FVC z-score > −1.65
Mild	FEV1/FVC ≥ 0.7 and −2.5 ≤ FVC z-score ≤ −1.65
Moderate	FEV1/FVC ≥ 0.7 and −4 ≤ FVC z-score < −2.5
Severe	FEV1/FVC ≥ 0.7 and FVC z-score < −4
GOLD grades of AFO	
GOLD 1	FEV1/FVC < 0.7 and FEV1 ≥ 80% predicted
GOLD 2	FEV1/FVC < 0.7 and 50% ≤ FEV1 < 80% predicted
GOLD 3	FEV1/FVC < 0.7 and 30% ≤ FEV1 < 50% predicted
GOLD 4	FEV1/FVC < 0.7 and FEV1 < 30% predicted

AFO = airflow obstruction; FEV1 = forced expiratory volume in 1 s; FVC = forced vital capacity; GOLD = Global Initiative for Chronic Obstructive Lung Disease; RSP = restrictive spirometric pattern.

### Assessment of covariates

Height, weight, and waist circumference (WC) were measured by trained staff following a standard protocol. BMI was calculated by dividing weight by the square of height (kg/m^2^). Based on the recommended criteria for Chinese adults^25^ and after combining groups with small sample size, we classified the participants’ body shape into three groups based on the joint grouping of BMI (kg/m^2^) and WC (female/male, cm): underweight (BMI < 18.5), normal (BMI 18.5–23.9 and WC < 80/85), and overweight/obesity or precentral/central obesity (BMI ≥ 24.0, or BMI 18.5–23.9 and WC ≥ 80/85; hereafter abbreviated as general or central obesity). The Supplementary methods section contains detailed descriptions of data collection and definitions of other covariates.

### Ascertainment of CVD outcomes

All participants were long-term followed up for their mortality, morbidity, and hospitalization events using a unique national identity linked to local death and disease registry systems and the national health insurance (HI) database. For those who failed to be linked to the HI database, annual active follow-up was conducted to identify the occurrence of major outcome events or moving out of the city. All diagnoses were coded by the International Classification of Diseases 10th version (ICD-10). Independent clinical adjudication of retrieved medical records for CVD cases is underway in CKB, as described in Supplementary methods.

The CVD outcomes of interest in this study included acute myocardial infarction (AMI), other ischaemic heart disease (other IHD) except AMI, heart failure, pulmonary heart disease, arrhythmia, ischaemic stroke (IS), intracerebral haemorrhage (ICH), and other cerebrovascular disease (other CBD) except stroke. The detailed ICD-10 codes for these outcomes are shown in Supplementary Table S2.

### Statistical analysis

We excluded the following participants from the total cohort of 512,723 participants (Supplementary Fig. S3): (1) those who reported having coronary heart disease (CHD) (n = 15,472, 3.0%) or stroke (n = 8884, 1.7%) at baseline; (2) ever smokers (n = 166,083, 32.4%); and (3) those with missing data on variables of interest (n = 2723, 0.5%). Given the substantial sex disparity in the number of never-smokers (278,953 females and 50,845 males, 84.6% and 15.4%, respectively), we analyzed and presented all results separately by sex, prioritizing female findings as the primary results.

The baseline characteristics of study participants, grouped by spirometric patterns, were described using percentages or means (standard deviation [SD]). Person-years were calculated from the baseline date until the date of the CVD outcome of interest, death, loss to follow-up, or December 31, 2018, whichever came first. In the association analyses of heart failure, pulmonary heart disease, and arrhythmia, those who had other CVD outcomes that occurred before the onset of these three outcomes were censored at the earliest incident date of other CVD outcomes.

With age as the time scale, we used stratified Cox proportional hazard models to estimate the hazard ratios (HRs) and 95% confidence intervals (CIs) of spirometric pattern with the eight CVD outcomes, stratified jointly by five-year age groups and ten survey areas. By referring to previous studies,^6^^,^^26^ multivariable models were adjusted for age, educational level, occupation, marital status, household income, alcohol consumption, physical activity, intakes of red meat, fresh fruits and vegetables, BMI, WC, fuel types used for cooking and heating, years of solid fuel use, stove ventilation, passive smoking, and family histories of heart disease and stroke. The severity of AFO and reduced FVC and the continuous FEV1%P and FVC percent predicted (FVC%P) were also included in models to test the dose–response effect. Plots of the scaled Schoenfeld residuals^27^ showed no evident violations of the proportional hazard assumption. The sex-interaction analysis was conducted using likelihood ratio test to compare models with and without the interaction term.

The following sensitivity analyses were performed among females: (1) excluding new CVD cases identified during the first five years of follow-up; (2) defining AFO as FEV1/FVC below the lower limit of normal (LLN)^28^ and RSP as FEV1/FVC ≥ LLN and FVC < LLN; (3) additionally adjusting for hypertension, diabetes, and the use of lipid-lowering medications (a proxy for dyslipidemia) at baseline; and (4) using the Fine and Gray model to assess the impact of competing events (deaths from causes other than the outcome of interest).

We further conducted subgroup analyses stratified by the following variables: age (<60 years, ≥60 years), area (urban, rural), and body shape (underweight, normal, general or central obesity). We applied Bonferroni-corrected significance thresholds of *P* < 0.0062 (0.05/8 outcomes). Statistical analyses were completed using Stata (StataCorp, version 17.0) and R (R Foundation for Statistical Computing, version 4.3.1).

### Role of the funding source

The funders had no role in the study design, data collection, data analysis and interpretation, writing of the report, or the decision to submit the article for publication.

## Results

### Baseline characteristics of study participants

Among 278,953 female participants, 156,219 (56.0%) lived in rural areas. The mean baseline age was 50.8 (SD, 10.2) years, with 63,997 (22.9%) having RSP and 11,327 (4.1%) having AFO. Among 50,845 male participants, 25,453 (50.1%) lived in rural areas. The mean baseline age was 50.4 (SD, 11.5) years, 14,548 (28.6%) had RSP, and 2421 (4.8%) had AFO. At baseline, both females and males with AFO were older and more likely from rural areas, but had lower BMI and WC than the other two groups, whereas those with RSP had higher BMI and WC and were more likely to use solid fuels for heating at baseline (Table 2). Furthermore, participants with RSP or AFO had lower FEV1%P and FVC%P, as well as a higher prevalence of respiratory symptoms than the normal group.

**Table 2:** Baseline characteristics of participants according to spirometric pattern.

Baseline characteristics	Female			Male		
	Normal	With RSP	With AFO	Normal	With RSP	With AFO
No. of participants	203,629	63,997	11,327	33,876	14,548	2421
Age, year	50.2 (9.9)	51.7 (10.7)	56.4 (11.2)	51.3 (11.3)	53.7 (11.5)	60.1 (10.9)
Rural, %	55.9	54.8	63.8	52.2	41.6	70.5
Below middle school, %	52.9	62.7	74.8	30.7	36.0	61.8
Industrial and related workers, %	12.3	9.2	6.1	18.5	18.8	7.4
Household income <20,000 Chinese Yuan last year, %	55.6	65.3	70.3	51.2	53.5	71.0
Married, %	90.6	88.6	83.6	93.8	92.0	85.7
Weekly alcohol drinking, %	1.9	1.3	2.0	22.5	18.8	18.5
Physical activity, MET-h/d	21.0 (12.9)	20.9 (12.7)	19.3 (12.5)	22.2 (15.2)	19.9 (13.9)	18.3 (14.7)
Regular consumption of						
Red meat, %	26.7	27.4	20.0	34.0	36.2	20.6
Fresh vegetables, %	95.9	90.1	95.3	96.3	91.1	95.7
Fresh fruits, %	22.0	20.6	14.6	18.5	19.0	11.6
Body mass index, kg/m^2^	23.8 (3.3)	24.0 (3.7)	22.9 (3.6)	23.8 (3.0)	24.1 (3.4)	22.4 (3.3)
Waist circumference, cm	78.4 (9.1)	80.4 (10.1)	77.1 (10.0)	82.4 (9.1)	84.5 (10.0)	78.8 (10.1)
Current cooking with solid fuels, %	47.7	52.3	58.6	17.2	12.9	29.4
Years of cooking with solid fuels, year	19.5 (16.2)	23.1 (18.1)	28.0 (18.2)	7.1 (13.5)	6.4 (13.8)	12.2 (19.0)
Stove ventilation, %	74.4	82.6	72.6	72.0	84.0	62.3
Current heating with solid fuels, %	33.9	45.6	28.8	33.7	37.6	37.3
Years of heating with solid fuels, year	16.9 (20.4)	22.9 (22.9)	14.5 (21.6)	18.2 (21.6)	21.4 (23.6)	20.8 (25.7)
Passive smoking^a^, %	60.8	60.3	58.8	55.7	50.4	45.8
Years lived with smokers, year	26.8 (18.0)	27.3 (18.8)	31.4 (20.6)	12.6 (14.7)	11.4 (14.7)	14.2 (17.0)
Hours exposed to smoke weekly, hour	6.2 (9.4)	5.7 (8.3)	5.8 (8.9)	5.3 (8.7)	4.8 (8.1)	4.3 (8.4)
Family history of						
Heart attack, %	3.3	3.1	2.6	3.2	3.2	2.1
Stroke, %	18.7	14.6	14.5	19.0	14.6	15.0
FEV1%P, %	99.7 (11.9)	73.4 (10.9)	65.3 (20.2)	98.9 (11.0)	74.5 (11.9)	61.9 (20.7)
FVC%P, %	97.6 (11.4)	69.4 (8.9)	86.1 (24.5)	95.7 (10.5)	68.5 (9.8)	79.7 (23.6)
Having respiratory symptoms^b^, %	10.2	13.8	25.1	10.2	11.9	25.9

AFO = airflow obstruction; FEV1%P = forced expiratory volume in 1 s (FEV1) percent predicted; FVC%P = forced vital capacity (FVC) percent predicted; MET = metabolic equivalent of task; RSP = restrictive spirometric pattern.

Values are percentages or means (standard deviation) and group comparisons were tested using ANOVA and chi-square tests. Except that the *P* value was 0.013 for family history of heart attack among males, the other *P* values were <0.001.

a Nonsmokers inhaling smoke from other smokers at least one day per week for ≥ 5 min each time.

b Respiratory symptoms include shortness of breath while walking, slowing down while walking due to chest discomfort, coughing frequently last year, and coughing up sputum frequently last year.

### Associations between spirometric pattern and CVD outcomes

During a median follow-up of 12 years (3,063,752 person-years), there were 3169 AMI, 24,948 other IHD, 2492 heart failure, 2004 pulmonary heart disease, 4355 arrhythmia, 25,351 IS, 4898 ICH, and 23,660 other CBD occurred among females. For heart failure, pulmonary heart disease, and arrhythmia, there were 2035, 1832, and 3808 cases remaining, respectively, after censoring at dates for any other incident CVD outcomes prior to the onset of these three CVD outcomes.

**Fig. 1:**
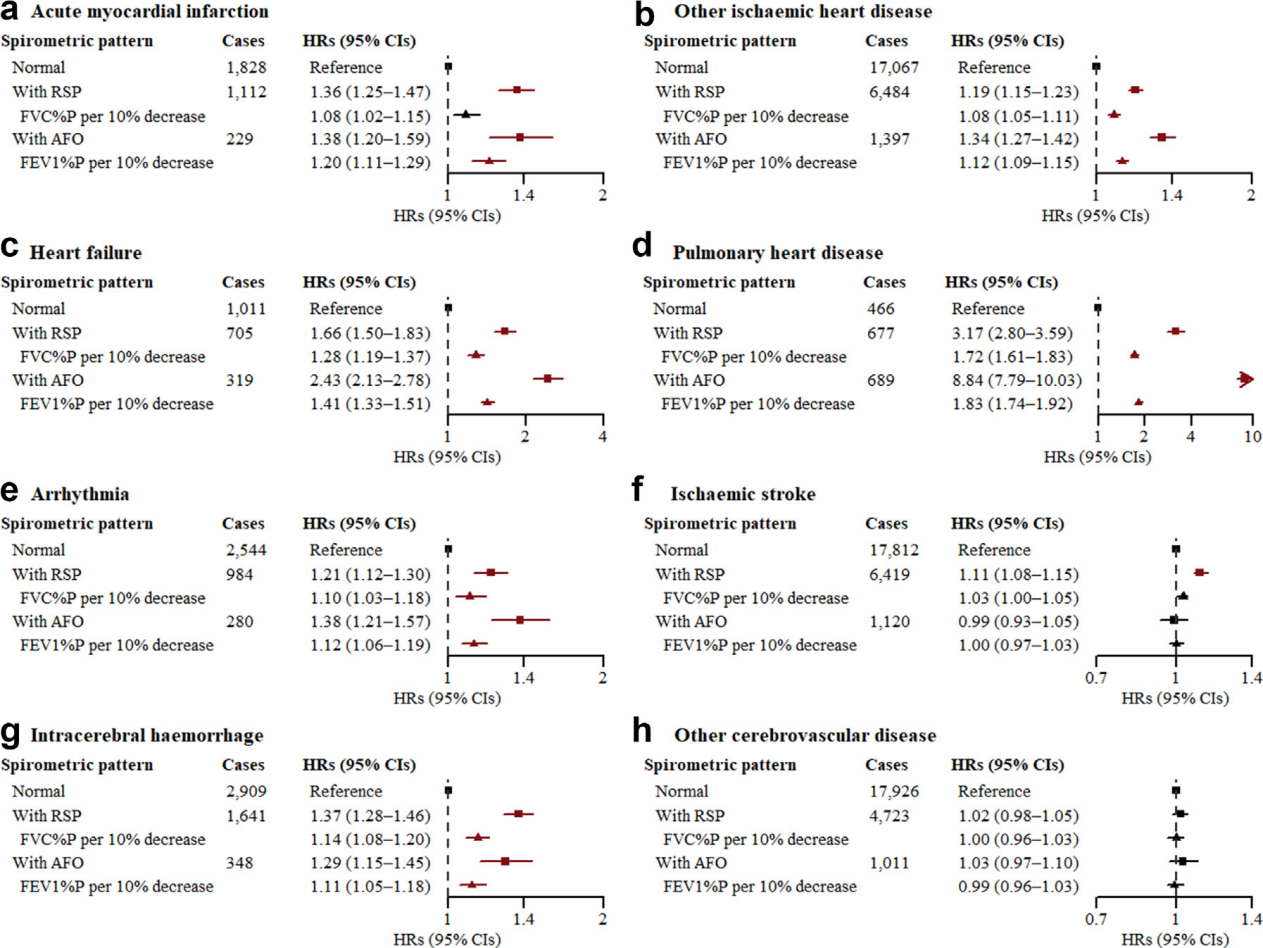
**Associations between spirometric pattern and CVD outcomes among females.** AFO = airflow obstruction; CI = confidence interval; CVD = cardiovascular disease; FEV1 = forced expiratory volume in 1 s; FEV1%P = FEV1 percent predicted; FVC = forced vital capacity; FVC%P = FVC percent predicted; HR = hazard ratio; RSP = restrictive spirometric pattern. “Normal” refers to FEV1/FVC ≥ 0.7 and FVC ≥ 80% predicted; “With RSP” refers to FEV1/FVC ≥ 0.7 and FVC < 80% predicted; “With AFO” refers to FEV1/FVC < 0.7. Analyses of FVC%P and FEV1%P were conducted among participants with RSP or AFO, respectively. The HRs (95% CIs) in red were statistically significant after Bonferroni correction (<0.0062). Multivariable models were adjusted for age, level of education, occupation, marital status, household income, alcohol consumption, physical activity, intake frequencies of red meat, fruits, and vegetables, body mass index, waist circumference, fuel types currently used in cooking, fuel types currently used in heating, years of cooking with solid fuels, years of heating with solid fuels, stove ventilation in the baseline house, passive smoking, and family histories of heart disease and stroke.

After adjusting for potential confounding factors, the presence of RSP was associated with increased risks of seven outcomes except other CBD among females, with HRs (95% CIs) of 1.36 (1.25–1.47) for AMI, 1.19 (1.15–1.23) for other IHD, 1.66 (1.50–1.83) for heart failure, 3.17 (2.80–3.59) for pulmonary heart disease, 1.21 (1.12–1.30) for arrhythmia, 1.11 (1.08–1.15) for IS, and 1.37 (1.28–1.46) for ICH (Fig. 1). The association strengths increased with the decline of FVC%P among females with RSP, with the risk rising by 8% for other IHD, 28% for heart failure, 72% for pulmonary heart disease, 10% for arrhythmia, and 14% for ICH per 10% decrease in FVC%P. The risks of six CVD outcomes other than IS and other CBD elevated among females with AFO, slightly higher than those with RSP, with HRs (95% CIs) of 1.38 (1.20–1.59) for AMI, 1.34 (1.27–1.42) for other IHD, 2.43 (2.13–2.78) for heart failure, 8.84 (7.79–10.03) for pulmonary heart disease, 1.38 (1.21–1.57) for arrhythmia, and 1.29 (1.15–1.45) for ICH. Similar dose–response relationships were observed in the associations between FEV1%P and the six outcomes among females with AFO, with the risk rising by 20% for AMI, 12% for other IHD, 41% for heart failure, 83% for pulmonary heart disease, 12% for arrhythmia, and 11% for ICH per 10% decrease in FEV1%P. Interaction tests revealed moderate differences between sexes in the associations between spirometric patterns and other CBD (*P*_*int*_ = 0.0002) (Supplementary Table S3). Males' results were mostly identical to females’, although the associations between spirometric patterns and arrhythmia were no longer statistically significant (Supplementary Fig. S4).

Sensitivity analyses of excluding CVD cases documented during the first five years of follow-up, defining the spirometric pattern using the LLN criterion, and additionally adjusting for hypertension, diabetes, and lipid-lowering medications use did not alter the females' results appreciably (Supplementary Table S4). The subdistribution hazard ratios (SHRs) of the competing risk models were generally consistent with the HRs of Cox models (Supplementary Table S5).

### Associations between severity of reduced FVC and AFO with CVD outcomes

**Fig. 2:**
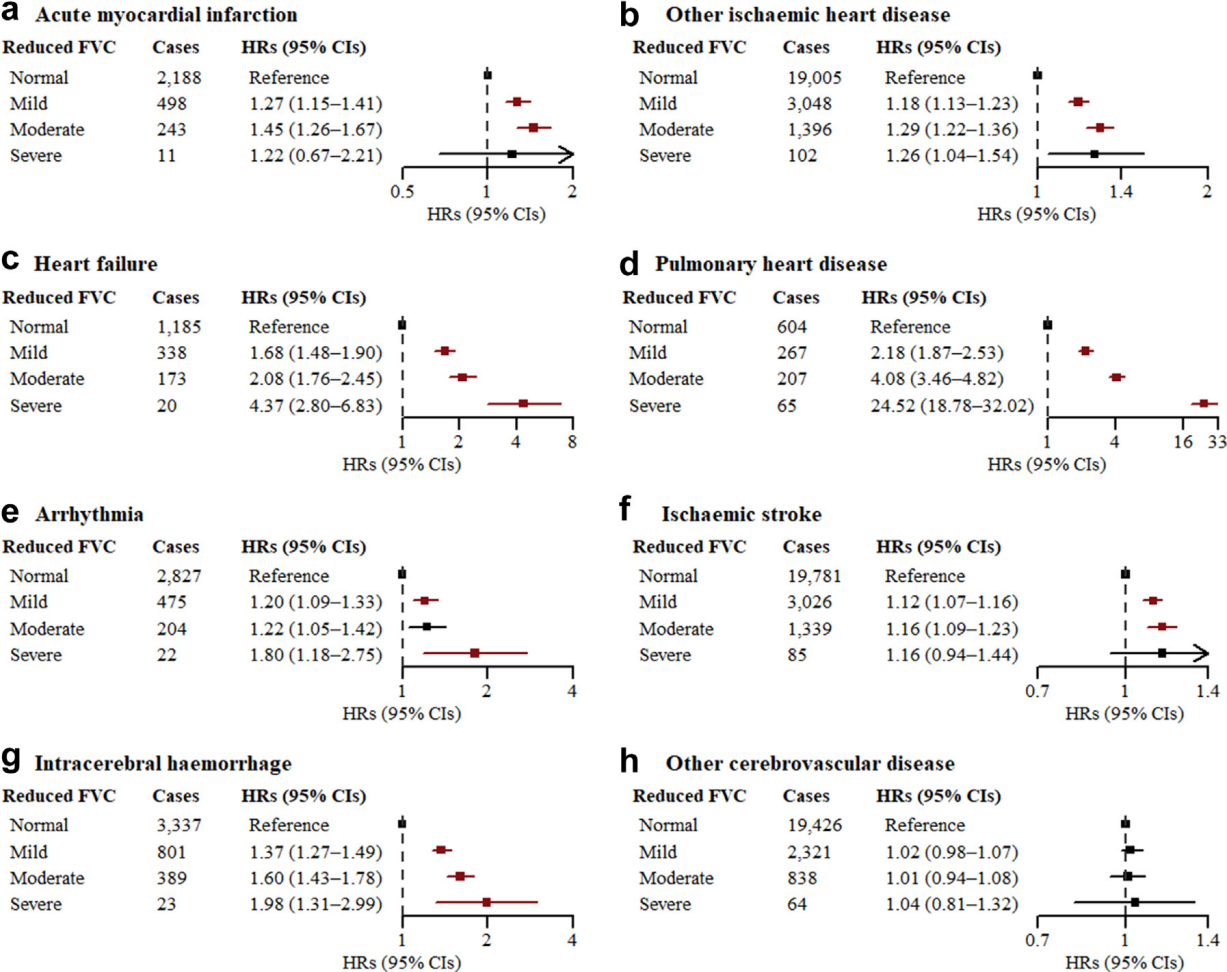
**Associations between severity of reduced FVC and CVD outcomes among females without AFO.** AFO = airflow obstruction; CI = confidence interval; CVD = cardiovascular disease; FEV1 = forced expiratory volume in 1 s; FVC = forced vital capacity; HR = hazard ratio. “Normal” refers to FEV1/FVC ≥ 0.7 and FVC z-score > −1.65; “Mild” refers to FEV1/FVC ≥ 0.7 and −2.5 ≤ FVC z-score ≤ −1.65; “Moderate” refers to FEV1/FVC ≥ 0.7 and −4 ≤ FVC z-score < −2.5; “Severe” refers to FEV1/FVC ≥ 0.7 and FVC z-score < −4. The HRs (95% CIs) in red were statistically significant after Bonferroni correction (<0.0062). Multivariable models were adjusted for age, level of education, occupation, marital status, household income, alcohol consumption, physical activity, intake frequencies of red meat, fruits, and vegetables, body mass index, waist circumference, fuel types currently used in cooking, fuel types currently used in heating, years of cooking with solid fuels, years of heating with solid fuels, stove ventilation in the baseline house, passive smoking, and family histories of heart disease and stroke.

**Fig. 3:**
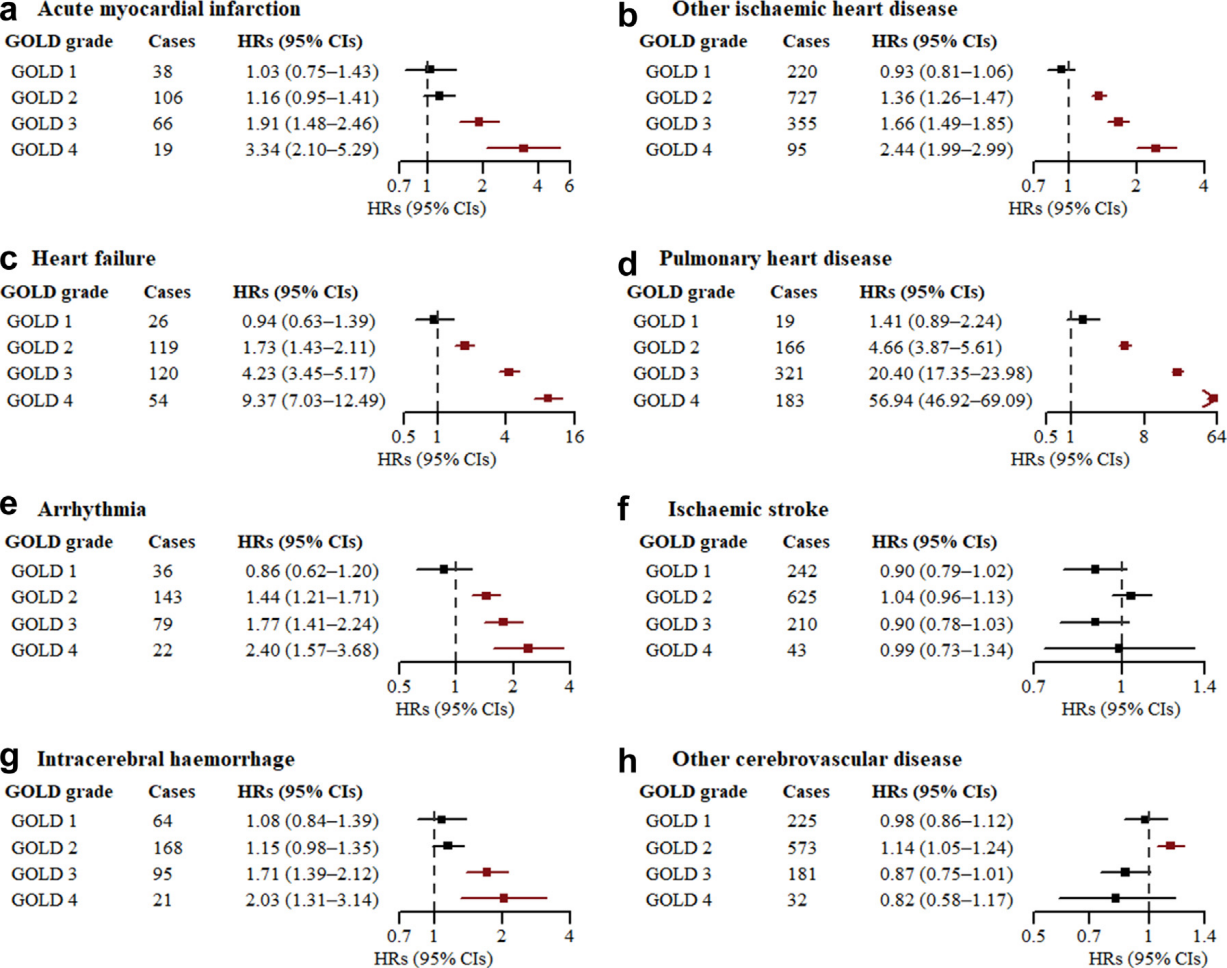
**Associations between GOLD grades of AFO and CVD outcomes among females without RSP.** AFO = airflow obstruction; CI = confidence interval; CVD = cardiovascular disease; FEV1 = forced expiratory volume in 1 s; FEV1%P = FEV1 percent predicted; FVC = forced vital capacity; GOLD = Global Initiative for Chronic Obstructive Lung Disease; HR = hazard ratio; RSP = restrictive spirometric pattern. “GOLD 1” refers to FEV1/FVC < 0.7 and FEV1%P ≥ 80%; “GOLD 2” refers to FEV1/FVC < 0.7 and 50% ≤ FEV1%P < 80%; “GOLD 3” refers to FEV1/FVC < 0.7 and 30% ≤ FEV1%P < 50%; “GOLD 4” refers to FEV1/FVC < 0.7 and FEV1%P < 30%. The HRs (95% CIs) in red were statistically significant after Bonferroni correction (<0.0062). Multivariable models took those with FEV1/FVC ≥ 0.7 and FVC ≥ 80% predicted (the “normal” group) as the reference, and were adjusted for age, level of education, occupation, marital status, household income, alcohol consumption, physical activity, intake frequencies of red meat, fruits, and vegetables, body mass index, waist circumference, fuel types currently used in cooking, fuel types currently used in heating, years of cooking with solid fuels, years of heating with solid fuels, stove ventilation in the baseline house, passive smoking, and family histories of heart disease and stroke.

Among participants without AFO, the risks of AMI, other IHD, heart failure, pulmonary heart disease, IS, and ICH increased with the severity of reduced FVC regardless of sex, with the highest HRs for pulmonary heart disease in mild, moderate, and severe groups being 2.18 (1.87–2.53), 4.08 (3.46–4.82), and 24.52 (18.78–32.02) among females (Fig. 2) and 1.75 (1.20–2.56), 5.07 (3.59–7.15), and 19.70 (12.04–32.23) among males (Supplementary Fig. S5). The gradient associations between reduced FVC z-score and arrhythmia was observed among females but not males. Compared to the normal group without RSP and AFO, there was a stepwise increasing association through GOLD grades with most CVD outcomes except IS and other CBD among females, with the HRs for GOLD 4 ranging from 2.03 (1.31–3.14) for ICH to 56.94 (46.92–69.09) for pulmonary heart disease (Fig. 3). Analyses conducted among males yielded comparable results for other IHD, heart failure, and pulmonary heart disease, although associations for other CVD outcomes diminished with wide CIs (Supplementary Fig. S6). The findings did not change materially with competing risks considered in Fine and Gray model (Supplementary Table S5).

### Subgroup analyses stratified by age, region, and body shape

**Fig. 4:**
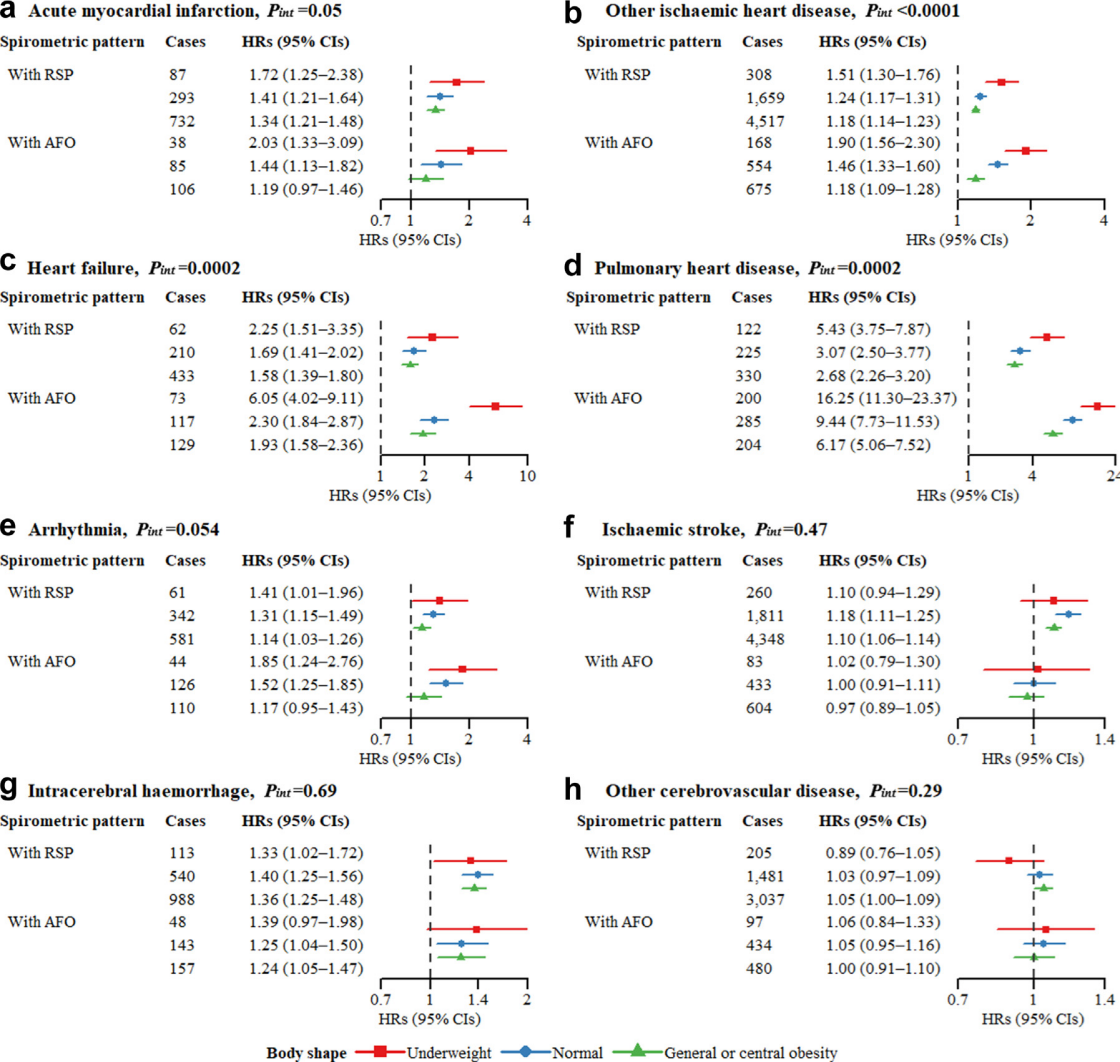
**Associations between spirometric pattern and CVD outcomes stratified by body shape among females.** AFO = airflow obstruction; BMI = body mass index; CI = confidence interval; CVD = cardiovascular disease; FEV1 = forced expiratory volume in 1 s; FVC = forced vital capacity; HR = hazard ratio; RSP = restrictive spirometric pattern; WC = waist circumference. In the spirometric pattern, “With RSP” refers to FEV1/FVC ≥ 0.7 and FVC < 80% predicted; “With AFO” refers to FEV1/FVC < 0.7. In the body shape, “Underweight” refers to BMI < 18.5 kg/m^2^; “Normal” refers to BMI 18.5–23.9 kg/m^2^ and WC < 80/85 cm; “General or central obesity” refers to BMI ≥ 24 kg/m^2^, or BMI 18.5–23.9 kg/m^2^ and WC ≥ 80/85 cm. Multivariable models took those with FEV1/FVC ≥ 0.7 and FVC ≥ 80% predicted (the “normal” group) as the reference, and were adjusted for age, level of education, occupation, marital status, household income, alcohol consumption, physical activity, intake frequencies of red meat, fruits, and vegetables, fuel types currently used in cooking, fuel types currently used in heating, years of cooking with solid fuels, years of heating with solid fuels, stove ventilation in the baseline house, passive smoking, and family histories of heart disease and stroke.

Among females, the associations of spirometric pattern with pulmonary heart disease (*P*_*int*_ = 0.002) and IS (*P*_*int*_ < 0.0001) were stronger in individuals aged <60 than in those aged ≥60 (Supplementary Table S6). Rural females had stronger associations of spirometric pattern with other IHD (*P*_*int*_ < 0.0001) and arrhythmia (*P*_*int*_ = 0.0039) than urban females (Supplementary Table S7). Furthermore, spirometric pattern had stronger associations with other IHD (*P*_*int*_ < 0.0001), heart failure (*P*_*int*_ = 0.0002), and pulmonary heart disease (*P*_*int*_ = 0.0002) in underweight females compared to normal and obese females (Fig. 4). The HRs of RSP with the three outcomes were 1.51 (1.30–1.76), 2.25 (1.51–3.35), and 5.43 (3.75–7.87) for underweight females and 1.18 (1.14–1.23), 1.58 (1.39–1.80), and 2.68 (2.26–3.20) for obese females. The HRs of AFO with the three outcomes were 1.90 (1.56–2.30), 6.05 (4.02–9.11), and 16.25 (11.30–23.37) for underweight females and 1.18 (1.09–1.28), 1.93 (1.58–2.36), and 6.17 (5.06–7.52) for obese females. These differences between subgroups mostly dissipated among males limited by less cases (Supplementary Fig. S7 and Tables S8 and S9).

## Discussion

In this large cohort study of only never-smokers, the presence of AFO was associated with increased risks of AMI, other IHD, heart failure, pulmonary heart disease, arrhythmia, and ICH, with the risks increasing with the GOLD grades of AFO. RSP and severity of reduced FVC were also associated with elevated risks of the six outcomes listed above, as well as IS. Females who were underweight and with RSP or AFO had higher risks of other IHD, heart failure, and pulmonary heart disease than their normal and obese counterparts.

Prior related studies on never-smokers used different exposures and outcomes, yielding fragmented evidence.^6^, ^7^, ^8^, ^9^ A Danish cohort of 26,005 never-smokers found no increased risks of hospitalization for IHD, MI, and heart failure in AFO patients compared to non-patients.^6^ A subgroup analysis of 332,496 never-smokers in another retrospective cohort also found no statistically significant association between ICD-diagnosed COPD and IHD.^7^ In the Atherosclerosis Risk in Communities (ARIC) cohort, IS risk was not linked to FEV1%P among both 4244 white and 1667 African American never-smokers.^8^ However, in 14,571 Swedish non-smokers, there was a negative association between FEV1/FVC quartiles and subarachnoid haemorrhage (SAH).^9^ The relatively small number of cases for each CVD outcome among never-smokers may have limited the statistical power of the above studies. With a large sample size, we identified positive associations between AFO and the risks of AMI, other IHD, heart failure, pulmonary heart disease, arrhythmia, and ICH, as well as dose–response relationships of the risks with FEV1%P and GOLD grades. However, despite the thousands of IS cases in our study, no statistically significant association was observed.

The observed associations between AFO and CVD are unrelated to smoking and may be explained by the following mechanisms^4^^,^^29^: (1) Overinflation of the lungs compresses the heart, impeding circulation and oxygenation. (2) Pulmonary inflammation “spills over” into the systemic circulation, causing endothelial damage and platelet hyperreactivity, hence promoting atherosclerosis, thrombosis, and microvascular disorders. (3) Hypoxic vasoconstriction in the lungs causes pulmonary hypertension, which leads to right-sided heart failure and decreased cardiac output. Notably, AFO was linked to all cardiac outcomes but only to ICH in the context of cerebrovascular disease. This suggests that the two conditions may be caused by different mechanisms, which requires additional confirmation through mechanism studies and causal evidence.

Three cohort studies examined the associations between RSP and CVD outcomes after adjusting for smoking status.^10^, ^11^, ^12^ Two of them observed positive associations between RSP and heart failure among older Americans^10^ and middle-aged African Americans,^12^ while one found associations with composite CVD (MI, CHD death, and IS) in the ARIC cohort.^11^ The current study adds new evidence to this issue that is more reliable and independent of smoking. Elevated risks of heart failure, AMI, other IHD, and IS in participants with RSP were echoed in this study. We also observed heightened risks of pulmonary heart disease, arrhythmia, and ICH in this group, which increased as the FVC z-score or FVC%P declined. Although the magnitude was slightly weaker than that of AFO patients, the CVD risk in this group is nevertheless cause for concern.

The mechanisms underlying RSP and CVD are poorly understood. Existing data suggest associations of RSP with left ventricular hypertrophy, diastolic dysfunction, greater pulmonary artery pressure, and right ventricular dysfunction.^1^^,^^12^ RSP likely represents distinct pathophysiologic alterations in cardiac function compared to AFO, which shows left ventricular underfilling. In addition, RSP/reduced FVC had an inverse relationship and arterial stiffness.^30^ Plausible explanations include inflammation-mediated myocardial/vascular damage, intermittent hypoxia, and large fluctuations of sympathetic tone and intrathoracic pressure.^1^^,^^30^

Interestingly, body shape modified the associations between abnormal spirometric patterns and cardiac-related outcomes, which is similar to the previously reported obesity paradox in COPD. The novel discovery is that this paradox was also observed in RSP. Previous studies have reported that lean COPD patients had a higher prevalence of sarcopenia,^31^ leading to more severe AFO and hyperinflation,^32^ as well as a clinical profile with a poor prognosis.^16^ Obesity may improve airflow through mechanisms similar to chest wall straps, and also provide greater metabolic reserves.^31^ Underweight RSP patients may have failed to reach peak lung function due to early development and nutritional problems.^33^ In addition, elderly RSP patients may suffer from sarcopenia, muscular weakness, and frailty.^34^ The findings suggested that the aberrant spirometric patterns in underweight participants should be addressed further.

In this study, we applied the same statistical analysis to data from a large prospective cohort study with long-term follow-up and examined the associations between spirometric patterns and multiple CVD outcomes. Because of the large number of sample size and CVD cases, the study was able to limit all analyses to never-smokers to minimize the influence of smoking while still allowing for an in-depth analysis across all severity grades and various subgroups. The wealth of information collected at baseline allowed the study to control for potential confounding effects of several shared risk factors for pulmonary and cardiovascular diseases. Furthermore, by linking to multiple data sources, as many CVD events as possible were captured during the follow-up period.

This study also has some limitations. First, bronchodilators were not used when measuring lung function, which may have misclassified some non-AFO participants as having AFO. Second, the spirometric pattern simply reflected the baseline state, and new patients may occur subsequently. Both of the aforementioned scenarios may result in associations that were attenuated toward the null hypothesis. Third, due to data collection limitations, the study did not assess the disease control or progression of patients with RSP or AFO. Fourth, other unmeasured variables, such as low birth weight, may result in confounding. Finally, the study only included Chinese participants; further validation is needed to examine whether these findings are generalized to other populations.

### Conclusion

In conclusion, this large cohort study of Chinese never-smokers revealed that both RSP and AFO were associated with elevated risks of various CVD outcomes, which increased with the severity of AFO and reduced FVC. These associations were even stronger in underweight individuals. Further research is needed to explore the non-smoking mechanisms that contribute to increased cardiovascular risk in the patients with aberrant spirometric patterns, especially RSP. In addition to tobacco cessation, effective clinical approaches are required to improve the prevention and management of cardiovascular risk in patients with pulmonary diseases. Furthermore, particular attention should be paid to underweight patients with lung disease.

## Contributors

JL and LL conceived and designed the study and contributed to the interpretation of the results and critical revision of the manuscript for valuable intellectual content. LL, ZC, and JC, as the members of the CKB steering committee, designed and supervised the conduct of the whole study, obtained funding, and together with CY, DiS, PP, LY, YC, HD, DaS, MB, and LZ, acquired the CKB data. YD and JH accessed, verified, and analyzed the data. YD drafted the manuscript. All authors had access to the data, have read and approved the final manuscript, and accept responsibility for the decision to submit for publication. The corresponding authors attest that all listed authors meet authorship criteria and that no others meeting the criteria have been omitted. JL and LL are the guarantors.

## Data sharing statement

Researchers who are interested in obtaining the raw data from the CKB study that underlines this paper should contact pdc@kscdc.net. Details of how to access CKB data are available at https://www.ckbiobank.org/site/data+access.

## Declaration of interests

We declare that we have no conflicts of interest.
