# Supplementary material for: Spirometric pattern and cardiovascular risk: a prospective study of 0.3 million Chinese never-smokers
Source: Lancet Reg Health West Pac. 2024 Dec 30;54:101274. doi: 10.1016/j.lanwpc.2024.101274 (PMC11750506; doi:10.1016/j.lanwpc.2024.101274)
Supplement: Supplementary Figs. S1–S7 and Tables S1–S9 [file mmc1.docx]

**Supplemental Material**

Supplemental material for the manuscript entitled ***Spirometric pattern and cardiovascular risk: a prospective study of 0.3 million Chinese never-smokers***

**Contents**

[Members of the China Kadoorie Biobank collaborative group 2](#_Toc186382023)

[Supplementary methods 3](#_Toc186382024)

[Supplementary Table S1. Characteristics of healthy non-smokers used to assess the lung function equations 5](#_Toc186382025)

[Supplementary Table S2. ICD-10 codes for CVD outcomes 6](#_Toc186382026)

[Supplementary Table S3. Associations between spirometric pattern and CVD outcomes stratified by sex 7](#_Toc186382027)

[Supplementary Table S4. Sensitivity analyses of associations between spirometric pattern and CVD outcomes among females 8](#_Toc186382028)

[Supplementary Table S5. Competing risk analyses of associations between spirometric pattern, GOLD grades, and severity of reduced FVC with CVD outcomes among females 9](#_Toc186382029)

[Supplementary Table S6. Associations between spirometric pattern and CVD outcomes among females stratified by age groups 10](#_Toc186382030)

[Supplementary Table S7. Associations between spirometric pattern and incident CVD outcomes among females stratified by region 11](#_Toc186382031)

[Supplementary Table S8. Associations between spirometric pattern and CVD outcomes among males stratified by age groups 12](#_Toc186382032)

[Supplementary Table S9. Associations between spirometric pattern and CVD outcomes among males stratified by region 13](#_Toc186382033)

[Supplementary Fig. S1: Raw (data points) and mean (connected points, in 2-year age intervals) values of FEV1 and FVC in 43,783 healthy non-smokers 14](#_Toc186382034)

[Supplementary Fig. S2: Evaluation of the lung function equations in 43,783 healthy non-smokers 15](#_Toc186382035)

[Supplementary Fig. S3: Study flow chart. 16](#_Toc186382036)

[Supplementary Fig. S4: Associations between spirometric pattern and CVD outcomes among males 17](#_Toc186382037)

[Supplementary Fig. S5: Associations between severity of reduced FVC and CVD outcomes among males without AFO 18](#_Toc186382038)

[Supplementary Fig. S6: Associations of GOLD grades of AFO and CVD outcomes among males without RSP 19](#_Toc186382039)

[Supplementary Fig. S7: Associations between spirometric pattern and CVD outcomes stratified by body shape among males 20](#_Toc186382040)

**Members of the China Kadoorie Biobank collaborative group**

**International Steering Committee:** Junshi Chen, Zhengming Chen (PI), Robert Clarke, Rory Collins, Liming Li (PI), Jun Lv, Richard Peto, Robin Walters.

**International Co-ordinating Centre, Oxford:** Daniel Avery, Maxim Barnard, Derrick Bennett, Ruth Boxall, Ka Hung Chan, Yiping Chen, Zhengming Chen, Charlotte Clarke, Johnathan Clarke; Robert Clarke, Huaidong Du, Ahmed Edris Mohamed, Hannah Fry, Simon Gilbert, Pek Kei Im, Andri Iona, Maria Kakkoura, Christiana Kartsonaki, Kshitij Kolhe, Hubert Lam, Kuang Lin, James Liu, Mohsen Mazidi, Iona Millwood, Sam Morris, Qunhua Nie, Alfred Pozarickij, Maryam Rahmati, Paul Ryder, Dan Schmidt, Becky Stevens, Iain Turnbull, Robin Walters, Baihan Wang, Lin Wang, Neil Wright, Ling Yang, Xiaoming Yang, Pang Yao.

**National Co-ordinating Centre, Beijing:** Jun Lv, Canqing Yu, Dianjianyi Sun, Yuanjie Pang, Can Hou, Qingmei Xia, Chao Liu, Pei Pei, Lang Pan, Xiao Han, Honglu Bian, Xinxin Chen.

**10 Regional Co-ordinating Centres: Qingdao CDC:** Zengchang Pang, Ruqin Gao, Shanpeng Li, Haiping Duan, Shaojie Wang, Yongmei Liu, Ranran Du, Liang Cheng, Xiaocao Tian, Hua Zhang. **Licang CDC:** Dan Hu, Xiaoyan Zheng, Yujie Wang. **Heilongjiang Provincial CDC:** Wei Sun, Shichun Yan, Xiaoming Cui. **Nangang CDC:** Chi Wang, Zhenyuan Wu, Lishun Zhai, Zhaoxi Pang, Shiwen Dong. **Hainan Provincial CDC:** Huiming Luo, Jinyan Chen, Bin He, Dingwei Sun, Xingren Wang, Tingting Ou. **Meilan CDC:** Xiangyang Zheng, Dewei Zheng, Shuai Yang, Yilei Li, Lihui Li, Xingjiao Chen. **Jiangsu Provincial CDC:** Jinyi Zhou, Ran Tao, Jian Su, Xikang Fan, Zongming Cheng, Yuxiao Huang. **Suzhou CDC:** Yan Lu, Yujie Hua, Li Xing, Shuxian Wang, Jianrong Jin, Juping Ma, Jinchao Liu, Kaifei Zhu, Hongfu Ren, Xingfeng Shen. **Guangxi Provincial CDC:** Ge Zhong, Wei Mao, Zhenzhen Lu, Ling He. **Liuzhou CDC:** Lifang Zhou, Changping Xie, Jian Lan, Tingping Zhu, Jinxue Tan, Liuping Wei, Liyuan Zhou, Sisi Wang. **Sichuan Provincial CDC:** Xianping Wu, Ningmei Zhang, Xiaofang Chen, Xiaoyu Chang, Zhuo Wang, Yujin He. **Pengzhou CDC:** Mingqiang Yuan, Xia Wu, Xiaofang Chen, Zhaodong Wang, Qiang Sun, Yang Lin. **Gansu Provincial CDC:** Faqing Chen, Xiaolan Ren, Lijun Chang, Feiming Zhong. **Maiji CDC:** Jianjun Feng, Weijie Hu, Xiaofang Zhang, Yalin Chen, Fei Wang, Jun Wang. **Henan Provincial CDC:** Linqi Diao, Wanshen Guo, Zhiwei Han, Dongyang Zhao, Dengjun Zhu, Kai Kang, Shixian Feng, Huizi Tian, Yali Yan, Bing Han, Li Gao, Shaofang Li, Huafei Feng, Wei Tang. **Huixian CDC:** Xiaolin Li, Huarong Sun, Xiaocong Zhao, Ying Li, Chen Hu, Pan He, Xukui Zhang, Yuanyuan Jin, Hesheng Zhang. **Zhejiang Provincial CDC:** Min Yu, Ruying Hu, Hao Wang, Weiwei Gong, Jieming Zhong, Meng Wang, Chunxiao Xu, Keqing Gong. **Tongxiang CDC**: Hao Xu, Yuan Cao, Kaixu Xie, Lingli Chen, Xiaomei Tu,Chen Chen. **Hunan Provincial CDC:** Xiaojun Li, Li Yin, Huilin Liu, Yuan Liu, Yi Liu, Lei Yin, Xian Xie, Jing Wang. **Liuyang CDC:** Bo Xiao, Pingsheng Lou, Yuan Peng, Libo Zhang, Chan Qu, Qili Jiang, Yanling Chen, Yan Zhao.

**Event Adjudication Clinicians:**

**Beijing Tiantan Hospital,Capital Medical University** Shuya Li, Haiqiang Qin, Yongjun Wang, **Peking University People's Hospital** Qiling Chen, Jihua Wang, **The 1^st^ Affiliated Hospital of Harbin Medical University** Xiaojia Sun, Lei Wang, Xun Wang, Liming Zhang, Shanshan Zhou, **The 2^nd^ Affiliated Hospital of Harbin Medical University** Hongyuan Chen, Li Chen, Haiyan Gou, Weizhi Wang, Yanmei Zhu, Yulan Zhu, **The 2^nd^ Hospital of Hebei Medical University** Ning Zhang, **Huashan Hospital** Xin Cheng, Qiang Dong, Yi Dong, Kun Fang, Yiting Mao, **Jinling Hospital** Yu An, Peiling Chen, Yinghua Chen, Zhihong Liu, Lihua Zhang **The People's Hospital of Liaoning Province** Xiaohong Chen, Naixin Jv, Xiaojiu Li, Liyang Liu, Yun Lu, Xiaona Xing, **Qingdao Fuwai Cardiovascular Hospital** Shihao You, **Shengjing Hospital of China Medical University** Xiaoli Cheng, Chaojun Gua, Jinping Jiang, Jingyi Liu, Shumei Ma, **Shenyang Military General Hospital** Xuefeng Yang, **The First People's Hospital of Shenyang** Xiaomo Du, Jian Xu, Xuecheng Yang, Xiaodi Zhao, **West China Hospital, Sichuan University** Zilong Hao, Ming Liu, Deren Wang, **The Second Affiliated Hospital of Suzhou University** Xiaoting Li, **Suzhou Kowloon Hospital Shanghai Jiao Tong University School of Medicine** Lili Hui, Zhanling Liao, Feng Liu, **Qingdao Fuwai Cardiovascular Hospital** Chunning Feng, Dejiang Ji, Fengxia Qu, Wenwen Yuan, **The First Affiliated Hospital of Zhengzhou University** Xin Fu, **Zhongshan Hospital,** Jing Ding, Peng Du, Lirong Jin, Yueshi Mao, Xin Wang.

**Supplementary methods**

**Selection criteria of healthy non-smokers used to assess the lung function equations**

(1) smoking <100 cigarettes in lifetime; (2) without self-reported respiratory system diseases including emphysema, bronchitis, asthma, and tuberculosis, as well as measured restrictive spirometric pattern (the ratio of forced expiratory volume in one second [FEV1] to forced vital capacity [FVC] ≥ 0.7 but FVC < 80% predicted); (3) no self-reported respiratory symptoms; (4) without exposure to wood or coal when cooking or heating for >5 years.

**Assessment of covariates**

Covariates obtained through the baseline questionnaire included sociodemographic characteristics (age, sex, education, occupation, income, and marital status), lifestyle habits (alcohol consumption, intake frequencies of red meat, fresh vegetables, and fresh fruits, and physical activity), household air pollution, passive smoking, and personal and family health history.

Participants were asked about their current or previous (quitters only) smoking habits, including frequency, type, and quantity. They were then categorized into four groups: never smokers, occasional smokers, ex-regular smokers, and current daily smokers, with the latter two being excluded from our study. Questions about alcohol consumption included typical drinking frequency, type of alcoholic beverage consumed habitually, and volume of alcohol consumed on a typical drinking day in the past 12 months. Physical activity data collection included information on usual types and durations of work, commute, household, and leisure activities in the past 12 months. Total daily physical activity was calculated by multiplying the metabolic equivalents (METs) of each activity by its number of hours spent, then summing all activity MET hours.^1^ We assessed habitual consumption frequencies (daily, 4–6 days per week, 1–3 days per week, monthly, never or rarely) of twelve common food categories in the past 12 months using a validated qualitative food frequency questionnaire.^2^ In the models, the midpoint value for each frequency group (7, 5, 2, 0.5, and 0 day per week, respectively) was used.

We asked each participant how long they had lived in their previous three households, as well as the type and frequency of fuel used in each household for cooking (less than monthly was considered not cooking regularly) and heating. When multiple fuels were used, only the most frequently used fuel was documented. Coal and wood were classified as "solid fuels", whereas natural gas, electricity, and central heating were classified as "clean fuels".^3^ Questions about ventilation were only asked for cooking stoves because heating was typically done with limited ventilation to maintain warmth. Participants were categorized based on the primary fuel type used in their baseline residence. Total exposure duration (in years) was calculated by combining the length of stay in each of the three households and the use of solid fuels.

Participants were also asked how many days per week, at home, work, or any other location, they inhaled smoke from other smokers for at least five minutes each time over the past year. Those who reported weekly exposure to secondhand smoke were further asked about their total hours exposed per week (h/week) and, if applicable, the length of time they lived with smokers (years).

The study questionnaire is available on the study’s website at [**https://www.ckbiobank.org/study-resources/survey-data**](https://www.ckbiobank.org/study-resources/survey-data).

**Reliability and reproducibility of main covariates**

Within a few weeks of the initial baseline survey of China Kadoorie Biobank, a quality control (QC) survey was conducted in 15,728 (3.1%) randomly selected participants, using a repeat questionnaire and measures on selected items.^4^ The mean period between baseline and QC survey is 17days. The baseline and QC survey had strong agreement on tobacco use and alcohol drinking, with the weighted κ coefficient being 0.94 and 0.79, respectively. The within-person Spearman correlation coefficients between baseline and QC measures were 0.84 for systolic blood pressure (SBP) and 0.77 for diastolic blood pressure (DBP).^4^

Following completion of the baseline survey in July 2008, a resurvey was undertaken in 10 study regions during August and October 2008, involving 19,802 (5%) of randomly chosen surviving participants. The height, weight, body mass index (BMI), and waist circumferences of resurvey showed high correlation with baseline measures, with the Spearman correlation coefficients being 0.99, 0.96, 0.93, and 0.84, respectively.^4^

**Case adjudication**

As of 31 December 2018, medical records of 30,974 newly documented ischemic heart disease (IHD) and 39,319 stroke cases had been retrieved and validated, with the diagnostic accuracy for IHD of 93.3%. In terms of stroke and its subtypes (ischaemic stroke [IS], subarachnoid haemorrhage [SAH], intracerebral haemorrhage [ICH]), the diagnostic accuracy was 96.3%, 91.2%, 94.5, and 92.5%, respectively, with the silent lacunar infarcts classified as IS using the new International Classification of Diseases (ICD-11) criteria.^5^

**Adjustment for multivariable models**

The models were adjusted for age (years), level of education (below middle school, middle school or high school, or college or higher), occupation (agriculture and related workers, factory workers, unemployed, or others), marital status (married, widowed, separated or divorced, or never married), household annual income (<10000 Chinese Yuan, 10000–19999 Chinese Yuan, or ≥20000 Chinese Yuan), alcohol consumption (less than weekly, former weekly, weekly but not daily, daily drinking <30, 30–59, or ≥60 g/day of pure alcohol), physical activity (MET-hours per day), intake frequencies of red meat, fruits, and vegetables (days per week), body mass index (kg/m^2^), waist circumference (cm), fuel types currently used in cooking (solid fuels, clean fuels, other fuels, or not cooking regularly), fuel types currently used in heating (solid fuels, clean fuels, other fuels, or no winter heating), years of cooking with solid fuels (years), years of heating with solid fuels (years), stove ventilation in the baseline house (having ventilation for all stoves, having ventilation but not for all stoves, no ventilation, no cooking facility), passive smoking (exposed <1day/week and <20y, <1day/week but ≥20y, ≥1day/week but <20y, ≥1day/week and ≥20y and <20h/week, or ≥1day/week and ≥20y and ≥20h/week), and family histories of heart disease and stroke (have or not have).

**Reference:**

1. Du H, Bennett D, Li L, Whitlock G, Guo Y, Collins R, et al. Physical activity and sedentary leisure time and their associations with BMI, waist circumference, and percentage body fat in 0.5 million adults: the China Kadoorie Biobank study. *Am J Clin Nutr*. 2013;**97**(3):487–96.

2. Han Y, Hu Y, Yu C, Guo Y, Pei P, Yang L, et al. Lifestyle, cardiometabolic disease, and multimorbidity in a prospective Chinese study. *Eur Heart J*. 2021;**42**(34):3374–84.

3. Wang S, Luo K. Life expectancy impacts due to heating energy utilization in China: Distribution, relations, and policy implications. *Sci Total Environ*. 2018;**610–611**:1047–56.

4. Chen Z, Chen J, Collins R, Guo Y, Peto R, Wu F, et al. China Kadoorie Biobank of 0.5 million people: survey methods, baseline characteristics and long-term follow-up. *Int J Epidemiol*. 2011;**40**(6):1652–66.

5. Turnbull I, Clarke R, Wright N, Guo Y, Kartsonaki C, Pei P, et al. Diagnostic accuracy of major stroke types in Chinese adults: A clinical adjudication study involving 40,000 stroke cases. *Lancet Reg Health West Pac*. 2022;**21**:100415.

**Supplementary Table S1. Characteristics of healthy non-smokers used to assess the lung function equations**

|  | Sex | Age (years) | N=43,783 | Height (cm) | Weight (kg) | BMI (kg/m^2^) | FEV1 (L) | FVC (L) | FEV1/FVC |
| --- | --- | --- | --- | --- | --- | --- | --- | --- | --- |
| North | Female | 30–39 | 2,142 | 159.8 (5.3) | 57.6 (8.1) | 22.5 (3.0) | 2.67 (0.32) | 3.02 (0.38) | 0.89 (0.05) |
|  |  | 40–49 | 3,189 | 159.5 (5.2) | 59.8 (8.3) | 23.5 (3.0) | 2.50 (0.30) | 2.84 (0.36) | 0.88 (0.05) |
|  |  | 50–59 | 2,028 | 157.8 (5.2) | 60.9 (8.7) | 24.5 (3.2) | 2.28 (0.30) | 2.62 (0.35) | 0.87 (0.05) |
|  |  | 60–69 | 915 | 154.9 (5.5) | 59.9 (9.1) | 24.9 (3.3) | 1.99 (0.30) | 2.36 (0.35) | 0.84 (0.05) |
|  |  | 70–79 | 276 | 152.1 (5.2) | 57.7 (8.8) | 24.9 (3.4) | 1.72 (0.27) | 2.08 (0.31) | 0.83 (0.06) |
|  | Male | 30–39 | 419 | 171.6 (5.9) | 72.3 (10.6) | 24.5 (3.1) | 3.62 (0.42) | 4.12 (0.49) | 0.88 (0.06) |
|  |  | 40–49 | 442 | 170.5 (6.0) | 72.0 (9.5) | 24.8 (3.0) | 3.41 (0.45) | 3.89 (0.49) | 0.88 (0.06) |
|  |  | 50–59 | 296 | 169.4 (6.3) | 71.6 (10.0) | 24.9 (3.0) | 3.12 (0.42) | 3.61 (0.48) | 0.87 (0.06) |
|  |  | 60–69 | 285 | 167.1 (5.9) | 67.2 (11.0) | 24.0 (3.2) | 2.75 (0.36) | 3.29 (0.44) | 0.84 (0.06) |
|  |  | 70–79 | 182 | 165.3 (6.3) | 64.0 (10.2) | 23.4 (3.1) | 2.49 (0.38) | 3.03 (0.45) | 0.83 (0.06) |
| South | Female | 30–39 | 7,126 | 156.0 (5.2) | 55.0 (7.7) | 22.6 (2.8) | 2.44 (0.30) | 2.83 (0.36) | 0.86 (0.06) |
|  |  | 40–49 | 11,618 | 155.9 (5.2) | 56.9 (8.0) | 23.4 (3.0) | 2.31 (0.29) | 2.70 (0.34) | 0.86 (0.05) |
|  |  | 50–59 | 6,597 | 154.6 (5.2) | 57.0 (8.2) | 23.8 (3.1) | 2.10 (0.28) | 2.46 (0.32) | 0.86 (0.05) |
|  |  | 60–69 | 2,184 | 152.1 (5.3) | 55.4 (8.4) | 23.9 (3.2) | 1.83 (0.26) | 2.17 (0.31) | 0.85 (0.06) |
|  |  | 70–79 | 561 | 150.6 (5.6) | 54.4 (8.6) | 24.0 (3.3) | 1.66 (0.25) | 1.96 (0.28) | 0.85 (0.06) |
|  | Male | 30–39 | 1,106 | 166.2 (5.9) | 65.7 (9.9) | 23.7 (3.0) | 3.32 (0.42) | 3.83 (0.49) | 0.87 (0.06) |
|  |  | 40–49 | 1,521 | 165.2 (5.9) | 65.1 (9.3) | 23.8 (2.8) | 3.11 (0.41) | 3.60 (0.47) | 0.87 (0.06) |
|  |  | 50–59 | 1,477 | 163.9 (5.9) | 64.3 (9.2) | 23.9 (2.9) | 2.82 (0.39) | 3.29 (0.44) | 0.86 (0.06) |
|  |  | 60–69 | 1,038 | 162.6 (6.0) | 62.4 (9.3) | 23.6 (2.9) | 2.55 (0.35) | 3.00 (0.40) | 0.85 (0.06) |
|  |  | 70–79 | 381 | 162.3 (6.0) | 61.3 (9.4) | 23.2 (2.9) | 2.32 (0.31) | 2.75 (0.37) | 0.85 (0.06) |

BMI=body mass index; FEV1=forced expiratory volume in 1 second; FVC=forced vital capacity.

Values are numbers of participants or means (standard deviation).

**Supplementary Table S2. ICD-10 codes for CVD outcomes**

| **Outcomes** | **ICD-10 codes** |
| --- | --- |
| Acute myocardial infarction | I21 |
| Other ischaemic heart disease | I20 and I22-I25 |
| Heart failure | I50 |
| Pulmonary heart disease | I26-I27 |
| Arrhythmia | I44-I49 |
| Ischaemic stroke | I63 |
| Intracerebral haemorrhage | I61 |
| Other cerebrovascular disease except stroke | I62 and I65-I69 |

CVD=cardiovascular disease; ICD-10=International Classification of Diseases (ICD) 10^th^ version.

**Supplementary Table S3. Associations between spirometric pattern and CVD outcomes stratified by sex**

|  | Female | |  | Male | | *P_int_* |
| --- | --- | --- | --- | --- | --- | --- |
|  | Cases | HRs (95% CIs) |  | Cases | HRs (95% CIs) |  |
| Acute myocardial infarction |  |  |  |  |  | 0.87 |
| Normal | 1,828 | Reference |  | 501 | Reference |  |
| With RSP | 1,112 | 1.36 (1.25–1.47) |  | 384 | 1.36 (1.18–1.58) |  |
| With AFO | 229 | 1.38 (1.20–1.59) |  | 83 | 1.39 (1.09–1.77) |  |
| Other ischaemic heart disease | |  |  |  |  | 0.19 |
| Normal | 17,067 | Reference |  | 2571 | Reference |  |
| With RSP | 6,484 | 1.19 (1.15–1.23) |  | 1464 | 1.23 (1.15–1.32) |  |
| With AFO | 1,397 | 1.34 (1.27–1.42) |  | 296 | 1.35 (1.19–1.53) |  |
| Heart failure |  |  |  |  |  | 0.53 |
| Normal | 1,011 | Reference |  | 170 | Reference |  |
| With RSP | 705 | 1.66 (1.50–1.83) |  | 174 | 1.77 (1.41–2.22) |  |
| With AFO | 319 | 2.43 (2.13–2.78) |  | 61 | 2.43 (1.79–3.31) |  |
| Pulmonary heart disease |  |  |  |  |  | 0.84 |
| Normal | 466 | Reference |  | 78 | Reference |  |
| With RSP | 677 | 3.17 (2.80–3.59) |  | 156 | 3.39 (2.54–4.53) |  |
| With AFO | 689 | 8.84 (7.79–10.03) | | 165 | 9.11 (6.82–12.17) |  |
| Arrhythmia |  |  |  |  |  | 0.56 |
| Normal | 2,544 | Reference |  | 440 | Reference |  |
| With RSP | 984 | 1.21 (1.12–1.30) |  | 227 | 1.15 (0.97–1.37) |  |
| With AFO | 280 | 1.38 (1.21–1.57) |  | 52 | 1.15 (0.85–1.56) |  |
| Ischaemic stroke |  |  |  |  |  | 0.013 |
| Normal | 17,812 | Reference |  | 3484 | Reference |  |
| With RSP | 6,419 | 1.11 (1.08–1.15) |  | 1896 | 1.20 (1.13–1.28) |  |
| With AFO | 1,120 | 0.99 (0.93–1.05) |  | 336 | 1.11 (0.98–1.24) |  |
| Intracerebral haemorrhage |  |  |  |  |  | 0.79 |
| Normal | 2,909 | Reference |  | 741 | Reference |  |
| With RSP | 1,641 | 1.37 (1.28–1.46) |  | 520 | 1.39 (1.23–1.57) |  |
| With AFO | 348 | 1.29 (1.15–1.45) |  | 119 | 1.24 (1.01–1.52) |  |
| Other cerebrovascular disease | |  |  |  |  | **0.0002** |
| Normal | 17,926 | Reference |  | 2804 | Reference |  |
| With RSP | 4,723 | 1.02 (0.98–1.05) |  | 1153 | 1.12 (1.04–1.20) |  |
| With AFO | 1,011 | 1.03 (0.97–1.10) |  | 226 | 0.93 (0.81–1.07) |  |

AFO=airflow obstruction; CI=confidence interval; CVD=cardiovascular disease; FEV1=forced expiratory volume in 1 second; FVC=forced vital capacity; HR=hazard ratio; RSP=restrictive spirometric pattern.

"Normal" refers to FEV1/FVC ≥ 0.7 and FVC ≥ 80% predicted; "With RSP" refers to FEV1/FVC ≥ 0.7 and FVC < 80% predicted; "With AFO" refers to FEV1/FVC < 0.7.

Multivariable models were adjusted for age, level of education, occupation, marital status, household income, alcohol consumption, physical activity, intake frequencies of red meat, fruits, and vegetables, body mass index, waist circumference, fuel types currently used in cooking, fuel types currently used in heating, years of cooking with solid fuels, years of heating with solid fuels, stove ventilation in the baseline house, passive smoking, and family histories of heart disease and stroke.

**Supplementary Table S4. Sensitivity analyses of associations between spirometric pattern and CVD outcomes among females**

|  | Sensitivity analysis 1 | Sensitivity analysis 2 | Sensitivity analysis 3 |
| --- | --- | --- | --- |
| Acute myocardial infarction |  |  |  |
| Normal | Reference | Reference | Reference |
| With RSP | **1.34 (1.22–1.47)** | **1.34 (1.23–1.46)** | **1.31 (1.21–1.42)** |
| FVC%P per 10% decrease | 1.04 (0.96–1.12) | 1.10 (1.02–1.20) | 1.08 (1.01–1.15) |
| With AFO | **1.38 (1.18–1.63)** | **1.29 (1.13–1.48)** | **1.37 (1.19–1.58)** |
| FEV1%P per 10% decrease | **1.20 (1.10–1.30)** | **1.20 (1.12–1.29)** | **1.20 (1.11–1.29)** |
| Other ischaemic heart disease |  |  |  |
| Normal | Reference | Reference | Reference |
| With RSP | **1.16 (1.12–1.20)** | **1.21 (1.17–1.25)** | **1.17 (1.13–1.21)** |
| FVC%P per 10% decrease | **1.05 (1.02–1.08)** | **1.07 (1.03–1.10)** | **1.08 (1.05–1.11)** |
| With AFO | **1.35 (1.26–1.44)** | **1.29 (1.22–1.36)** | **1.34 (1.26–1.42)** |
| FEV1%P per 10% decrease | **1.13 (1.09–1.16)** | **1.12 (1.09–1.15)** | **1.12 (1.09–1.15)** |
| Heart failure |  |  |  |
| Normal | Reference | Reference | Reference |
| With RSP | **1.56 (1.40–1.74)** | **1.79 (1.61–2.00)** | **1.63 (1.47–1.80)** |
| FVC%P per 10% decrease | **1.22 (1.13–1.32)** | **1.22 (1.11–1.35)** | **1.28 (1.19–1.37)** |
| With AFO | **2.27 (1.97–2.63)** | **2.29 (2.02–2.59)** | **2.43 (2.13–2.77)** |
| FEV1%P per 10% decrease | **1.40 (1.31–1.50)** | **1.42 (1.34–1.51)** | **1.41 (1.33–1.51)** |
| Pulmonary heart disease |  |  |  |
| Normal | Reference | Reference | Reference |
| With RSP | **3.07 (2.66–3.54)** | **3.07 (2.71–3.47)** | **3.18 (2.81–3.60)** |
| FVC%P per 10% decrease | **1.69 (1.57–1.82)** | **1.81 (1.67–1.96)** | **1.72 (1.61–1.83)** |
| With AFO | **8.13 (7.03–9.42)** | **6.91 (6.16–7.76)** | **8.84 (7.79–10.04)** |
| FEV1%P per 10% decrease | **1.76 (1.66–1.86)** | **1.87 (1.78–1.96)** | **1.83 (1.74–1.93)** |
| Arrhythmia |  |  |  |
| Normal | Reference | Reference | Reference |
| With RSP | **1.19 (1.09–1.30)** | **1.23 (1.13–1.35)** | **1.20 (1.11–1.30)** |
| FVC%P per 10% decrease | **1.10 (1.02–1.18)** | **1.10 (1.01–1.20)** | **1.10 (1.03–1.18)** |
| With AFO | **1.39 (1.20–1.60)** | **1.39 (1.23–1.55)** | **1.38 (1.21–1.57)** |
| FEV1%P per 10% decrease | **1.11 (1.04–1.19)** | **1.10 (1.04–1.16)** | **1.12 (1.05–1.19)** |
| Ischaemic stroke |  |  |  |
| Normal | Reference | Reference | Reference |
| With RSP | **1.10 (1.06–1.14)** | **1.13 (1.09–1.17)** | **1.08 (1.05–1.11)** |
| FVC%P per 10% decrease | 1.02 (0.98–1.05) | 1.03 (0.99–1.07) | 1.03 (1.00–1.05) |
| With AFO | 0.99 (0.93–1.07) | 0.99 (0.94–1.05) | 0.98 (0.92–1.04) |
| FEV1%P per 10% decrease | 1.00 (0.96–1.03) | 0.99 (0.97–1.02) | 1.00 (0.97–1.03) |
| Intracerebral haemorrhage |  |  |  |
| Normal | Reference | Reference | Reference |
| With RSP | **1.28 (1.19–1.39)** | **1.46 (1.36–1.57)** | **1.31 (1.23–1.40)** |
| FVC%P per 10% decrease | **1.14 (1.07–1.21)** | **1.10 (1.03–1.18)** | **1.13 (1.08–1.19)** |
| With AFO | **1.24 (1.08–1.43)** | **1.29 (1.16–1.43)** | **1.27 (1.13–1.42)** |
| FEV1%P per 10% decrease | **1.11 (1.04–1.19)** | **1.09 (1.04–1.15)** | **1.10 (1.04–1.17)** |
| Other cerebrovascular disease |  |  |  |
| Normal | Reference | Reference | Reference |
| With RSP | 1.01 (0.97–1.04) | 1.02 (0.98–1.06) | 1.00 (0.97–1.04) |
| FVC%P per 10% decrease | 1.00 (0.96–1.04) | 1.01 (0.97–1.06) | 1.00 (0.96–1.03) |
| With AFO | 1.05 (0.98–1.13) | 1.02 (0.97–1.08) | 1.03 (0.96–1.10) |
| FEV1%P per 10% decrease | 0.98 (0.95–1.02) | 1.00 (0.98–1.03) | 0.99 (0.96–1.02) |

AFO=airflow obstruction; CI=confidence interval; CVD=cardiovascular disease; FEV1=forced expiratory volume in 1 second; FEV1%P=FEV1 percent predicted; FVC=forced vital capacity; FVC%P=FVC percent predicted; HR=hazard ratio; RSP=restrictive spirometric pattern.

The values were HRs (95% CIs), with bolded ones being statistically significant after Bonferroni correction (<0.0062). "Normal" refers to FEV1/FVC ≥ 0.7 and FVC ≥ 80% predicted; "With RSP" refers to FEV1/FVC ≥ 0.7 and FVC < 80% predicted; "With AFO" refers to FEV1/FVC < 0.7.

Analyses of FVC%P and FEV1%P were conducted among participants with RSP or AFO, respectively. The first sensitivity analysis was conduct after excluding new CVD cases identified during the first five years of follow-up. The second sensitivity analysis defined AFO as FEV1/FVC below the lower limit of normal (LLN), RSP as FEV1/FVC ≥ LLN and FVC < LLN, and normal as FEV1/FVC ≥ LLN and FVC ≥ LLN. In the first two sensitivity analyses, multivariable models were adjusted for age, level of education, occupation, marital status, household income, alcohol consumption, physical activity, intake frequencies of red meat, fruits, and vegetables, body mass index, waist circumference, fuel types currently used in cooking, fuel types currently used in heating, years of cooking with solid fuels, years of heating with solid fuels, stove ventilation in the baseline house, passive smoking, and family histories of heart disease and stroke. The third sensitivity analysis additionally adjusted the models for hypertension, diabetes, and the use of lipid-lowering medications (a proxy for dyslipidemia) at baseline.

**Supplementary Table S5. Competing risk analyses of associations between spirometric pattern, GOLD grades, and severity of reduced FVC with CVD outcomes among females**

|  | Spirometric pattern | SHRs (95% CIs) |  | GOLD grades | SHRs (95% CIs) |  | Reduced FVC | SHRs (95% CIs) |
| --- | --- | --- | --- | --- | --- | --- | --- | --- |
| Acute myocardial infarction | Normal | Reference |  | Normal | Reference |  | Normal | Reference |
|  | With RSP | 1.31 (1.21–1.42) |  | GOLD 1 | 1.06 (0.77–1.46) |  | Mild | 1.23 (1.11–1.36) |
|  | With AFO | 1.30 (1.13–1.50) |  | GOLD 2 | 1.12 (0.91–1.37) |  | Moderate | 1.36 (1.18–1.56) |
|  |  |  |  | GOLD 3 | 1.61 (1.24–2.08) |  | Severe | 1.08 (0.59–1.96) |
|  |  |  |  | GOLD 4 | 2.33 (1.46–3.72) |  |  |  |
| Other ischaemic heart disease | Normal | Reference |  | Normal | Reference |  | Normal | Reference |
|  | With RSP | 1.17 (1.13–1.20) |  | GOLD 1 | 0.93 (0.81–1.06) |  | Mild | 1.16 (1.11–1.20) |
|  | With AFO | 1.28 (1.21–1.36) |  | GOLD 2 | 1.34 (1.24–1.45) |  | Moderate | 1.24 (1.17–1.31) |
|  |  |  |  | GOLD 3 | 1.47 (1.32–1.64) |  | Severe | 1.17 (0.95–1.42) |
|  |  |  |  | GOLD 4 | 1.74 (1.41–2.15) |  |  |  |
| Heart failure | Normal | Reference |  | Normal | Reference |  | Normal | Reference |
|  | With RSP | 1.59 (1.43–1.76) |  | GOLD 1 | 0.94 (0.63–1.39) |  | Mild | 1.62 (1.43–1.84) |
|  | With AFO | 2.29 (2.00–2.62) |  | GOLD 2 | 1.72 (1.41–2.09) |  | Moderate | 1.91 (1.62–2.26) |
|  |  |  |  | GOLD 3 | 3.65 (2.96–4.49) |  | Severe | 3.68 (2.33–5.80) |
|  |  |  |  | GOLD 4 | 6.69 (4.94–9.07) |  |  |  |
| Pulmonary heart disease | Normal | Reference |  | Normal | Reference |  | Normal | Reference |
|  | With RSP | 3.07 (2.70–3.49) |  | GOLD 1 | 1.43 (0.90–2.28) |  | Mild | 2.11 (1.81–2.46) |
|  | With AFO | 8.53 (7.51–9.69) |  | GOLD 2 | 4.63 (3.85–5.57) |  | Moderate | 3.81 (3.20–4.52) |
|  |  |  |  | GOLD 3 | 18.82 (15.93–22.22) |  | Severe | 22.36 (16.89–29.60) |
|  |  |  |  | GOLD 4 | 48.96 (39.74–60.32) |  |  |  |
| Arrhythmia | Normal | Reference |  | Normal | Reference |  | Normal | Reference |
|  | With RSP | 1.18 (1.09–1.27) |  | GOLD 1 | 0.86 (0.62–1.19) |  | Mild | 1.18 (1.07–1.30) |
|  | With AFO | 1.30 (1.15–1.48) |  | GOLD 2 | 1.41 (1.19–1.68) |  | Moderate | 1.17 (1.01–1.36) |
|  |  |  |  | GOLD 3 | 1.54 (1.22–1.95) |  | Severe | 1.63 (1.07–2.48) |
|  |  |  |  | GOLD 4 | 1.76 (1.14–2.71) |  |  |  |
| Ischaemic stroke | Normal | Reference |  | Normal | Reference |  | Normal | Reference |
|  | With RSP | 1.08 (1.05–1.12) |  | GOLD 1 | 0.90 (0.79–1.02) |  | Mild | 1.09 (1.05–1.14) |
|  | With AFO | 0.95 (0.89–1.01) |  | GOLD 2 | 1.03 (0.95–1.12) |  | Moderate | 1.10 (1.04–1.16) |
|  |  |  |  | GOLD 3 | 0.80 (0.69–0.92) |  | Severe | 1.05 (0.84–1.30) |
|  |  |  |  | GOLD 4 | 0.74 (0.55–1.00) |  |  |  |
| Intracerebral haemorrhage | Normal | Reference |  | Normal | Reference |  | Normal | Reference |
|  | With RSP | 1.33 (1.24–1.42) |  | GOLD 1 | 1.08 (0.84–1.39) |  | Mild | 1.34 (1.23–1.45) |
|  | With AFO | 1.23 (1.09–1.38) |  | GOLD 2 | 1.12 (0.96–1.31) |  | Moderate | 1.51 (1.36–1.69) |
|  |  |  |  | GOLD 3 | 1.51 (1.21–1.87) |  | Severe | 1.76 (1.16–2.66) |
|  |  |  |  | GOLD 4 | 1.53 (0.99–2.38) |  |  |  |
| Other cerebrovascular disease | Normal | Reference |  | Normal | Reference |  | Normal | Reference |
|  | With RSP | 0.99 (0.96–1.02) |  | GOLD 1 | 0.99 (0.87–1.13) |  | Mild | 1.00 (0.96–1.05) |
|  | With AFO | 0.98 (0.92–1.04) |  | GOLD 2 | 1.12 (1.03–1.22) |  | Moderate | 0.95 (0.89–1.02) |
|  |  |  |  | GOLD 3 | 0.75 (0.64–0.87) |  | Severe | 0.92 (0.72–1.18) |
|  |  |  |  | GOLD 4 | 0.59 (0.42–0.84) |  |  |  |

AFO=airflow obstruction; CI=confidence interval; CVD=cardiovascular disease; FEV1%P=forced expiratory volume in 1 second (FEV1) percent predicted; FVC=forced vital capacity; FVC%P=FVC percent predicted; GOLD=Global Initiative for Chronic Obstructive Lung Disease; RSP=restrictive spirometric pattern; SHR=subdistribution hazard ratio.

In the spirometric pattern, "Normal" refers to FEV1/FVC ≥ 0.7 and FVC ≥ 80% predicted; "With RSP" refers to FEV1/FVC ≥ 0.7 and FVC < 80% predicted; "With AFO" refers to FEV1/FVC < 0.7.

In the GOLD grades, "Normal" refers to FEV1/FVC ≥ 0.7 and FVC ≥ 80% predicted; "GOLD 1" refers to FEV1/FVC < 0.7 and FEV1%P ≥ 80%; "GOLD 2" refers to FEV1/FVC < 0.7 and 50% ≤ FEV1%P < 80%; "GOLD 3" refers to FEV1/FVC < 0.7 and 30% ≤ FEV1%P < 50%; "GOLD 4" refers to FEV1/FVC < 0.7 and FEV1%P < 30%.

In the severity of reduced FVC, "Normal" refers to FEV1/FVC ≥ 0.7 and FVC z-score > –1.65; "Mild" refers to FEV1/FVC ≥ 0.7 and –2.5 ≤ FVC z-score ≤ –1.65; "Moderate" refers to FEV1/FVC ≥ 0.7 and –4 ≤ FVC z-score < –2.5; "Severe" refers to FEV1/FVC ≥ 0.7 and FVC z-score < –4.

Multivariable models were adjusted for age, level of education, occupation, marital status, household income, alcohol consumption, physical activity, intake frequencies of red meat, fruits, and vegetables, body mass index, waist circumference, fuel types currently used in cooking, fuel types currently used in heating, years of cooking with solid fuels, years of heating with solid fuels, stove ventilation in the baseline house, passive smoking, and family histories of heart disease and stroke.

**Supplementary Table S6. Associations between spirometric pattern and CVD outcomes among females stratified by age groups**

|  | < 60 years | |  | ≥ 60 years | | *P_int_* |
| --- | --- | --- | --- | --- | --- | --- |
|  | Cases | HRs (95% CIs) |  | Cases | HRs (95% CIs) |  |
| Acute myocardial infarction |  |  |  |  |  | 0.039 |
| Normal | 733 | Reference |  | 1,095 | Reference |  |
| With RSP | 409 | 1.42 (1.24–1.62) |  | 703 | 1.31 (1.18–1.45) |  |
| With AFO | 61 | 1.79 (1.37–2.34) |  | 168 | 1.21 (1.03–1.43) |  |
| Other ischaemic heart disease |  |  |  |  |  | 0.24 |
| Normal | 10,748 | Reference |  | 6,319 | Reference |  |
| With RSP | 3,442 | 1.15 (1.10–1.20) |  | 3,042 | 1.22 (1.17–1.28) |  |
| With AFO | 490 | 1.32 (1.20–1.45) |  | 907 | 1.28 (1.20–1.38) |  |
| Heart failure |  |  |  |  |  | 0.1 |
| Normal | 442 | Reference |  | 569 | Reference |  |
| With RSP | 284 | 1.77 (1.51–2.08) |  | 421 | 1.53 (1.34–1.74) |  |
| With AFO | 90 | 2.79 (2.20–3.53) |  | 229 | 2.17 (1.85–2.55) |  |
| Pulmonary heart disease |  |  |  |  |  | **0.002** |
| Normal | 210 | Reference |  | 256 | Reference |  |
| With RSP | 264 | 3.38 (2.79–4.10) |  | 413 | 2.90 (2.47–3.41) |  |
| With AFO | 226 | 11.96 (9.79–14.61) |  | 463 | 7.08 (6.03–8.32) |  |
| Arrhythmia |  |  |  |  |  | 0.7 |
| Normal | 1,689 | Reference |  | 855 | Reference |  |
| With RSP | 556 | 1.15 (1.04–1.28) |  | 428 | 1.25 (1.11–1.41) |  |
| With AFO | 97 | 1.30 (1.05–1.60) |  | 183 | 1.35 (1.14–1.60) |  |
| Ischaemic stroke |  |  |  |  |  | **<0.0001** |
| Normal | 10,695 | Reference |  | 7,117 | Reference |  |
| With RSP | 3,541 | 1.14 (1.10–1.19) |  | 2,878 | 1.06 (1.01–1.11) |  |
| With AFO | 412 | 1.03 (0.93–1.14) |  | 708 | 0.91 (0.84–0.99) |  |
| Intracerebral haemorrhage |  |  |  |  |  | 0.57 |
| Normal | 1,649 | Reference |  | 1,260 | Reference |  |
| With RSP | 798 | 1.40 (1.28–1.54) |  | 843 | 1.33 (1.22–1.46) |  |
| With AFO | 100 | 1.26 (1.03–1.55) |  | 248 | 1.28 (1.11–1.47) |  |
| Other cerebrovascular disease |  |  |  |  |  | 0.0079 |
| Normal | 12,663 | Reference |  | 5,263 | Reference |  |
| With RSP | 2,918 | 1.02 (0.98–1.06) |  | 1,805 | 0.98 (0.93–1.04) |  |
| With AFO | 478 | 1.08 (0.99–1.19) |  | 533 | 0.89 (0.81–0.98) |  |

AFO=airflow obstruction; CI=confidence interval; CVD=cardiovascular disease; FEV1=forced expiratory volume in 1 second; FVC=forced vital capacity; HR=hazard ratio; RSP=restrictive spirometric pattern.

"Normal" refers to FEV1/FVC ≥ 0.7 and FVC ≥ 80% predicted; "With RSP" refers to FEV1/FVC ≥ 0.7 and FVC < 80% predicted; "With AFO" refers to FEV1/FVC < 0.7.

Multivariable models were adjusted for level of education, occupation, marital status, household income, alcohol consumption, physical activity, intake frequencies of red meat, fruits, and vegetables, body mass index, waist circumference, fuel types currently used in cooking, fuel types currently used in heating, years of cooking with solid fuels, years of heating with solid fuels, stove ventilation in the baseline house, passive smoking, and family histories of heart disease and stroke.

**Supplementary Table S7. Associations between spirometric pattern and incident CVD outcomes among females stratified by region**

|  | Urban | |  | Rural | | *P_int_* |
| --- | --- | --- | --- | --- | --- | --- |
|  | Cases | HRs (95% CIs) |  | Cases | HRs (95% CIs) |  |
| Acute myocardial infarction |  |  |  |  |  | 0.28 |
| Normal | 720 | Reference |  | 1,108 | Reference |  |
| With RSP | 456 | 1.28 (1.13–1.44) |  | 656 | 1.43 (1.28–1.59) |  |
| With AFO | 60 | 1.30 (0.99–1.70) |  | 169 | 1.42 (1.20–1.68) |  |
| Other ischaemic heart disease |  |  |  |  |  | **<0.0001** |
| Normal | 9,466 | Reference |  | 7,601 | Reference |  |
| With RSP | 3,623 | 1.14 (1.10–1.19) |  | 2,861 | 1.27 (1.21–1.33) |  |
| With AFO | 433 | 1.14 (1.03–1.26) |  | 964 | 1.49 (1.38–1.59) |  |
| Heart failure |  |  |  |  |  | 0.059 |
| Normal | 383 | Reference |  | 628 | Reference |  |
| With RSP | 242 | 1.63 (1.38–1.93) |  | 463 | 1.68 (1.48–1.91) |  |
| With AFO | 66 | 1.86 (1.42–2.44) |  | 253 | 2.67 (2.29–3.12) |  |
| Pulmonary heart disease |  |  |  |  |  | 0.017 |
| Normal | 85 | Reference |  | 381 | Reference |  |
| With RSP | 79 | 2.12 (1.54–2.91) |  | 598 | 3.41 (2.98–3.90) |  |
| With AFO | 59 | 8.98 (6.28–12.83) |  | 630 | 9.00 (7.85–10.31) |  |
| Arrhythmia |  |  |  |  |  | **0.0039** |
| Normal | 1,307 | Reference |  | 1,237 | Reference |  |
| With RSP | 454 | 1.11 (0.99–1.24) |  | 530 | 1.32 (1.19–1.48) |  |
| With AFO | 72 | 1.08 (0.85–1.38) |  | 208 | 1.57 (1.35–1.84) |  |
| Ischaemic stroke |  |  |  |  |  | 0.61 |
| Normal | 9,291 | Reference |  | 8,521 | Reference |  |
| With RSP | 3,511 | 1.11 (1.07–1.16) |  | 2,908 | 1.11 (1.06–1.16) |  |
| With AFO | 435 | 0.95 (0.86–1.04) |  | 685 | 1.02 (0.94–1.10) |  |
| Intracerebral haemorrhage |  |  |  |  |  | 0.074 |
| Normal | 820 | Reference |  | 2,089 | Reference |  |
| With RSP | 406 | 1.38 (1.22–1.56) |  | 1,235 | 1.37 (1.27–1.48) |  |
| With AFO | 56 | 0.98 (0.75–1.30) |  | 292 | 1.37 (1.20–1.56) |  |
| Other cerebrovascular disease |  |  |  |  |  | 0.47 |
| Normal | 7,231 | Reference |  | 10,695 | Reference |  |
| With RSP | 2,029 | 1.03 (0.97–1.08) |  | 2,694 | 1.01 (0.96–1.06) |  |
| With AFO | 268 | 0.97 (0.85–1.09) |  | 743 | 1.06 (0.98–1.15) |  |

AFO=airflow obstruction; CI=confidence interval; CVD=cardiovascular disease; FEV1=forced expiratory volume in 1 second; FVC=forced vital capacity; HR=hazard ratio; RSP=restrictive spirometric pattern.

"Normal" refers to FEV1/FVC ≥ 0.7 and FVC ≥ 80% predicted; "With RSP" refers to FEV1/FVC ≥ 0.7 and FVC < 80% predicted; "With AFO" refers to FEV1/FVC < 0.7.

Multivariable models were adjusted for age, level of education, occupation, marital status, household income, alcohol consumption, physical activity, intake frequencies of red meat, fruits, and vegetables, body mass index, waist circumference, fuel types currently used in cooking, fuel types currently used in heating, years of cooking with solid fuels, years of heating with solid fuels, stove ventilation in the baseline house, passive smoking, and family histories of heart disease and stroke.

**Supplementary Table S8. Associations between spirometric pattern and CVD outcomes among males stratified by age groups**

|  | < 60 years | |  | ≥ 60 years | | *P_int_* |
| --- | --- | --- | --- | --- | --- | --- |
|  | Cases | HRs (95% CIs) |  | Cases | HRs (95% CIs) |  |
| Acute myocardial infarction | | |  |  |  | 0.59 |
| Normal | 175 | Reference |  | 326 | Reference |  |
| With RSP | 123 | 1.21 (0.94–1.57) |  | 261 | 1.45 (1.21–1.73) |  |
| With AFO | 14 | 1.60 (0.92–2.80) |  | 69 | 1.32 (1.01–1.72) |  |
| Other ischaemic heart disease | | |  |  |  | 0.55 |
| Normal | 1,232 | Reference |  | 1,339 | Reference |  |
| With RSP | 598 | 1.18 (1.06–1.31) |  | 866 | 1.27 (1.16–1.39) |  |
| With AFO | 65 | 1.37 (1.06–1.77) |  | 231 | 1.28 (1.11–1.48) |  |
| Heart failure |  |  |  |  |  | 0.007 |
| Normal | 47 | Reference |  | 123 | Reference |  |
| With RSP | 47 | 2.12 (1.37–3.29) |  | 127 | 1.61 (1.24–2.09) |  |
| With AFO | 19 | 5.18 (2.94–9.13) |  | 42 | 1.89 (1.31–2.71) |  |
| Pulmonary heart disease | |  |  |  |  | **0.0008** |
| Normal | 24 | Reference |  | 54 | Reference |  |
| With RSP | 50 | 4.55 (2.69–7.67) |  | 106 | 2.76 (1.96–3.89) |  |
| With AFO | 53 | 20.42 (12.17–34.25) |  | 112 | 6.28 (4.48–8.83) |  |
| Arrhythmia |  |  |  |  |  | 0.073 |
| Normal | 205 | Reference |  | 235 | Reference |  |
| With RSP | 105 | 1.29 (1.00–1.67) |  | 122 | 1.00 (0.80–1.26) |  |
| With AFO | 16 | 1.62 (0.96–2.74) |  | 36 | 0.91 (0.63–1.31) |  |
| Ischaemic stroke |  |  |  |  |  | 0.48 |
| Normal | 1,555 | Reference |  | 1,929 | Reference |  |
| With RSP | 755 | 1.21 (1.10–1.34) |  | 1,141 | 1.17 (1.08–1.27) |  |
| With AFO | 66 | 1.05 (0.81–1.35) |  | 270 | 1.07 (0.94–1.22) |  |
| Intracerebral haemorrhage | | |  |  |  | 0.96 |
| Normal | 351 | Reference |  | 390 | Reference |  |
| With RSP | 201 | 1.43 (1.18–1.73) |  | 319 | 1.35 (1.15–1.58) |  |
| With AFO | 26 | 1.40 (0.93–2.10) |  | 93 | 1.19 (0.94–1.50) |  |
| Other cerebrovascular disease | | |  |  |  | 0.11 |
| Normal | 1,502 | Reference |  | 1,302 | Reference |  |
| With RSP | 487 | 1.07 (0.96–1.19) |  | 666 | 1.13 (1.02–1.24) |  |
| With AFO | 70 | 1.09 (0.86–1.40) |  | 156 | 0.81 (0.68–0.96) |  |

AFO=airflow obstruction; CI=confidence interval; CVD=cardiovascular disease; FEV1=forced expiratory volume in 1 second; FVC=forced vital capacity; HR=hazard ratio; RSP=restrictive spirometric pattern.

"Normal" refers to FEV1/FVC ≥ 0.7 and FVC ≥ 80% predicted; "With RSP" refers to FEV1/FVC ≥ 0.7 and FVC < 80% predicted; "With AFO" refers to FEV1/FVC < 0.7.

Multivariable models were adjusted for level of education, occupation, marital status, household income, alcohol consumption, physical activity, intake frequencies of red meat, fruits, and vegetables, body mass index, waist circumference, fuel types currently used in cooking, fuel types currently used in heating, years of cooking with solid fuels, years of heating with solid fuels, stove ventilation in the baseline house, passive smoking, and family histories of heart disease and stroke.

**Supplementary Table S9. Associations between spirometric pattern and CVD outcomes among males stratified by region**

|  | Urban | |  | Rural | | *P_int_* |
| --- | --- | --- | --- | --- | --- | --- |
|  | Cases | HRs (95% CIs) |  | Cases | HRs (95% CIs) |  |
| Acute myocardial infarction | |  |  |  |  | 0.74 |
| Normal | 233 | Reference |  | 268 | Reference |  |
| With RSP | 215 | 1.32 (1.08–1.60) |  | 169 | 1.41 (1.13–1.75) |  |
| With AFO | 18 | 1.24 (0.76–2.02) |  | 65 | 1.42 (1.07–1.88) |  |
| Other ischaemic heart disease | |  |  |  |  | 0.29 |
| Normal | 1,516 | Reference |  | 1,055 | Reference |  |
| With RSP | 970 | 1.21 (1.12–1.32) |  | 494 | 1.26 (1.12–1.42) |  |
| With AFO | 88 | 1.19 (0.96–1.48) |  | 208 | 1.42 (1.21–1.66) |  |
| Heart failure |  |  |  |  |  | 0.99 |
| Normal | 83 | Reference |  | 87 | Reference |  |
| With RSP | 85 | 1.79 (1.30–2.46) |  | 89 | 1.81 (1.31–2.49) |  |
| With AFO | 18 | 2.46 (1.45–4.15) |  | 43 | 2.40 (1.63–3.53) |  |
| Pulmonary heart disease | |  |  |  |  | 0.76 |
| Normal | 14 | Reference |  | 64 | Reference |  |
| With RSP | 23 | 2.92 (1.45–5.89) |  | 133 | 3.49 (2.54–4.81) |  |
| With AFO | 14 | 13.40 (5.90–30.44) |  | 151 | 8.85 (6.48–12.10) |  |
| Arrhythmia |  |  |  |  |  | 0.73 |
| Normal | 234 | Reference |  | 206 | Reference |  |
| With RSP | 128 | 1.23 (0.98–1.54) |  | 99 | 1.07 (0.82–1.38) |  |
| With AFO | 13 | 1.05 (0.60–1.86) |  | 39 | 1.17 (0.81–1.67) |  |
| Ischaemic stroke |  |  |  |  |  | 0.56 |
| Normal | 1,980 | Reference |  | 1,504 | Reference |  |
| With RSP | 1,259 | 1.18 (1.10–1.27) |  | 637 | 1.25 (1.13–1.39) |  |
| With AFO | 123 | 1.15 (0.96–1.38) |  | 213 | 1.09 (0.94–1.27) |  |
| Intracerebral haemorrhage | |  |  |  |  | 0.94 |
| Normal | 255 | Reference |  | 486 | Reference |  |
| With RSP | 194 | 1.40 (1.15–1.71) |  | 326 | 1.38 (1.18–1.61) |  |
| With AFO | 22 | 1.32 (0.84–2.06) |  | 97 | 1.24 (0.99–1.56) |  |
| Other cerebrovascular disease | |  |  |  |  | 0.76 |
| Normal | 1,281 | Reference |  | 1,523 | Reference |  |
| With RSP | 648 | 1.12 (1.01–1.23) |  | 505 | 1.10 (0.99–1.23) |  |
| With AFO | 65 | 0.99 (0.77–1.28) |  | 161 | 0.91 (0.77–1.08) |  |

AFO=airflow obstruction; CI=confidence interval; CVD=cardiovascular disease; FEV1=forced expiratory volume in 1 second; FVC=forced vital capacity; HR=hazard ratio; RSP=restrictive spirometric pattern.

"Normal" refers to FEV1/FVC ≥ 0.7 and FVC ≥ 80% predicted; "With RSP" refers to FEV1/FVC ≥ 0.7 and FVC < 80% predicted; "With AFO" refers to FEV1/FVC < 0.7.

Multivariable models were adjusted for age, level of education, occupation, marital status, household income, alcohol consumption, physical activity, intake frequencies of red meat, fruits, and vegetables, body mass index, waist circumference, fuel types currently used in cooking, fuel types currently used in heating, years of cooking with solid fuels, years of heating with solid fuels, stove ventilation in the baseline house, passive smoking, and family histories of heart disease and stroke.


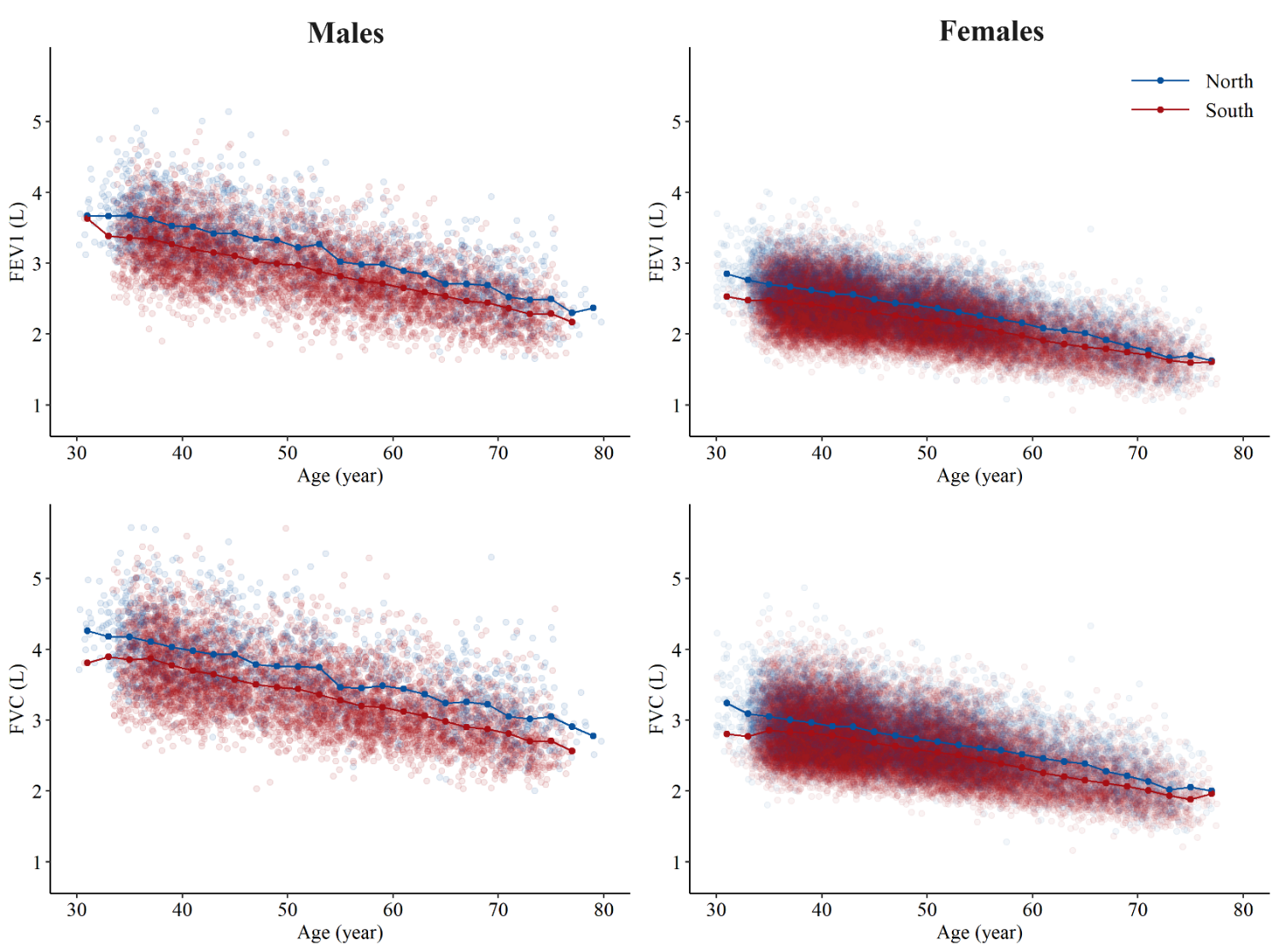


**Supplementary Fig. S1: Raw (data points) and mean (connected points, in 2-year age intervals) values of FEV1 and FVC in** **43,783 healthy non-smokers**

FEV1=forced expiratory volume in 1 second; FVC=forced vital capacity.


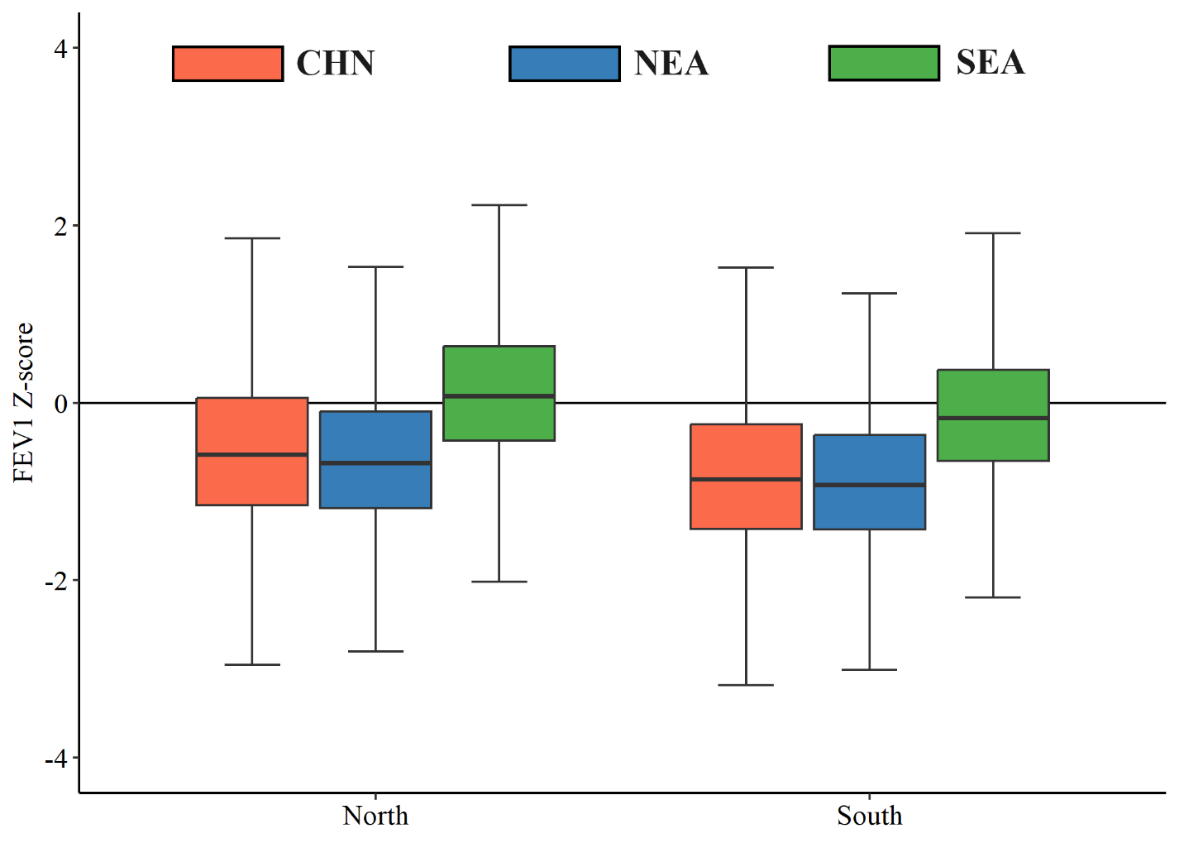


**Supplementary Fig. S2: Evaluation of the lung function equations in 43,783 healthy non-smokers**

FEV1=forced expiratory volume in 1 second.

"CHN" refers to the Chinese reference values; "NEA" and "SEA" refer to the Global Lung Function Initiative 2012 equations for South East Asian and North East Asian.

Values outside the whiskers were excluded.


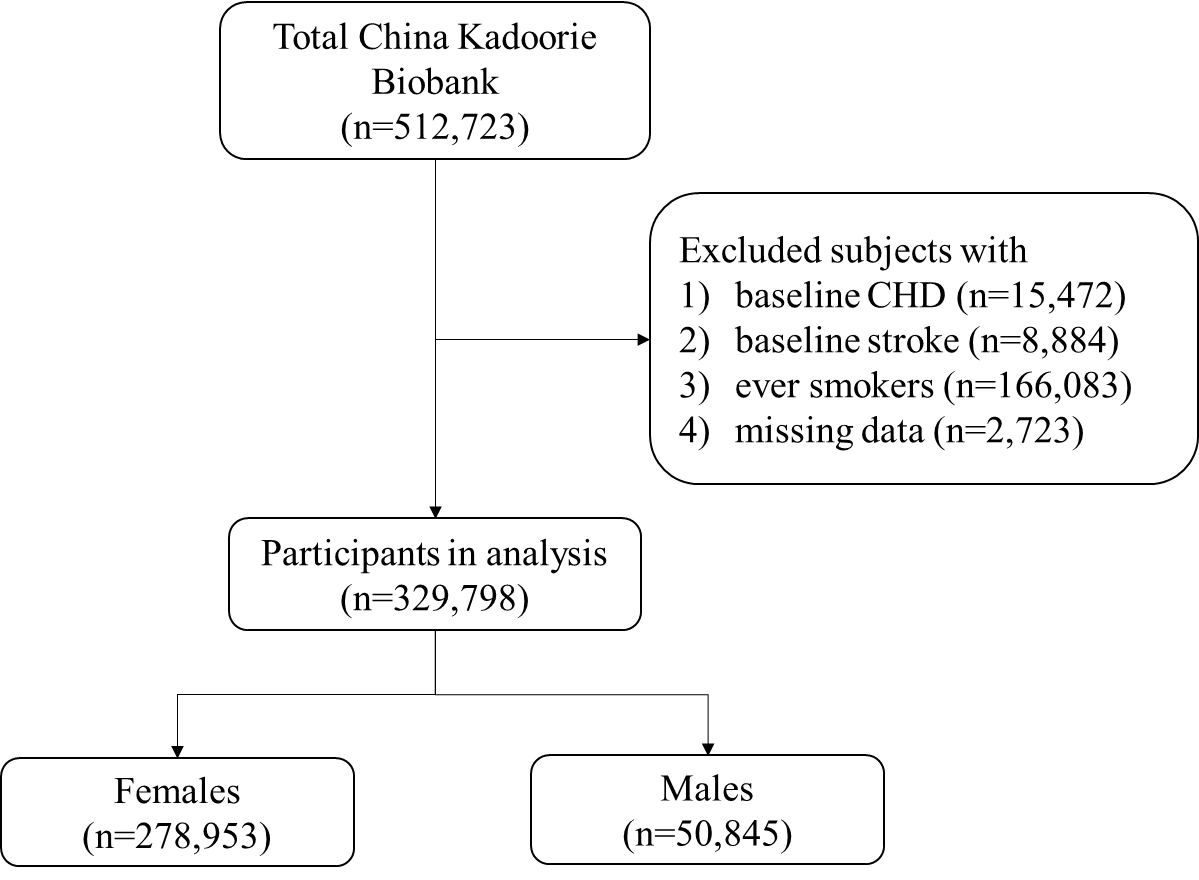


**Supplementary Fig. S3: Study flow chart.**

CHD=coronary heart disease.

**
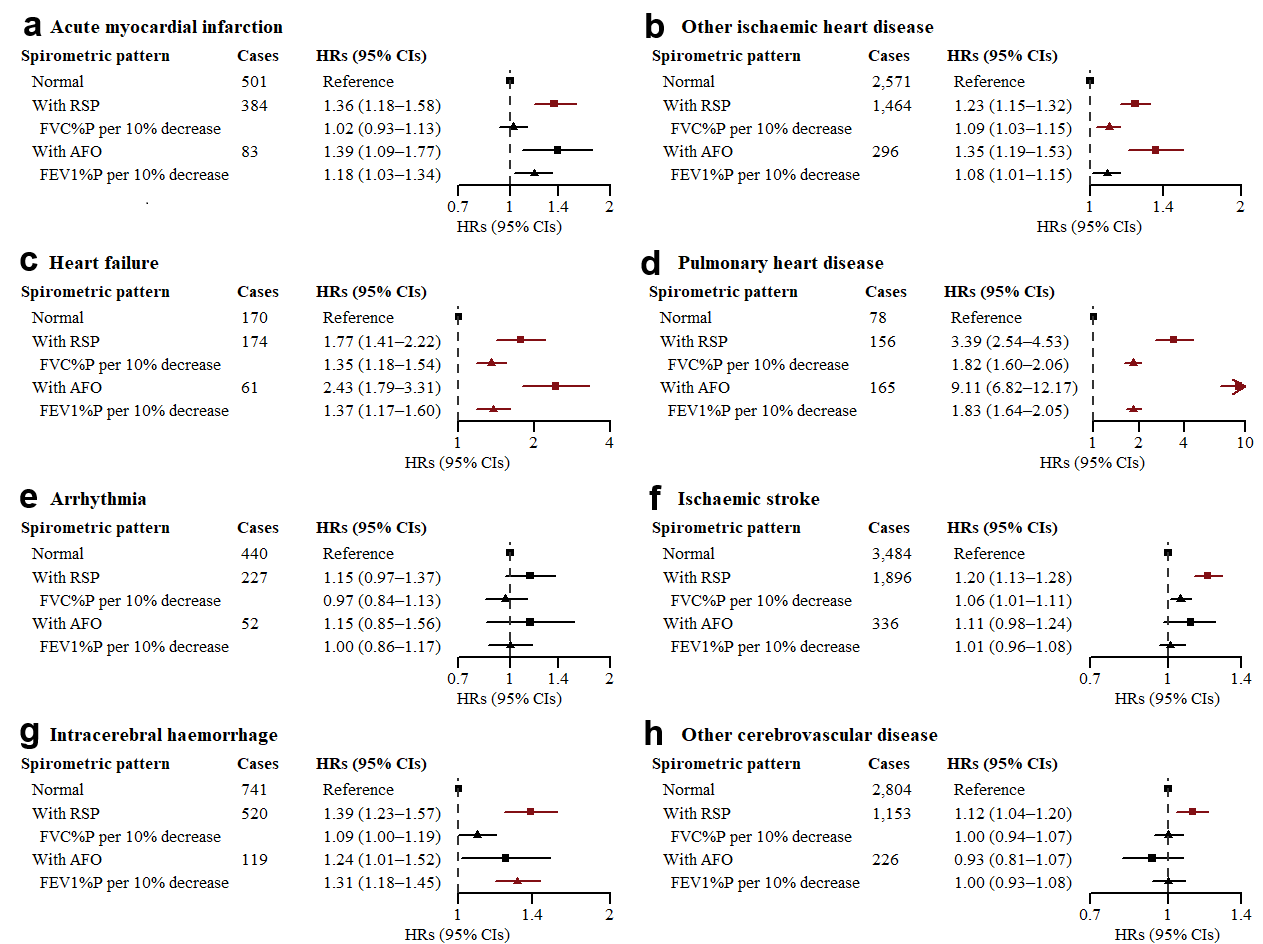
**

**Supplementary Fig. S4: Associations between spirometric pattern and CVD outcomes among males**

AFO=airflow obstruction; CI=confidence interval; CVD=cardiovascular disease; FEV1=forced expiratory volume in 1 second; FEV1%P=FEV1 percent predicted; FVC=forced vital capacity; FVC%P=FVC percent predicted; HR=hazard ratio; RSP=restrictive spirometric pattern.

"Normal" refers to FEV1/FVC ≥ 0.7 and FVC ≥ 80% predicted; "With RSP" refers to FEV1/FVC ≥ 0.7 and FVC < 80% predicted; "With AFO" refers to FEV1/FVC < 0.7.

Analyses of FVC%P and FEV1%P were conducted among participants with RSP or AFO patients, respectively. The HRs (95% CIs) in red were statistically significant after Bonferroni correction (<0.0062). Multivariable models were adjusted for age, level of education, occupation, marital status, household income, alcohol consumption, physical activity, intake frequencies of red meat, fruits, and vegetables, body mass index, waist circumference, fuel types currently used in cooking, fuel types currently used in heating, years of cooking with solid fuels, years of heating with solid fuels, stove ventilation in the baseline house, passive smoking, and family histories of heart disease and stroke.


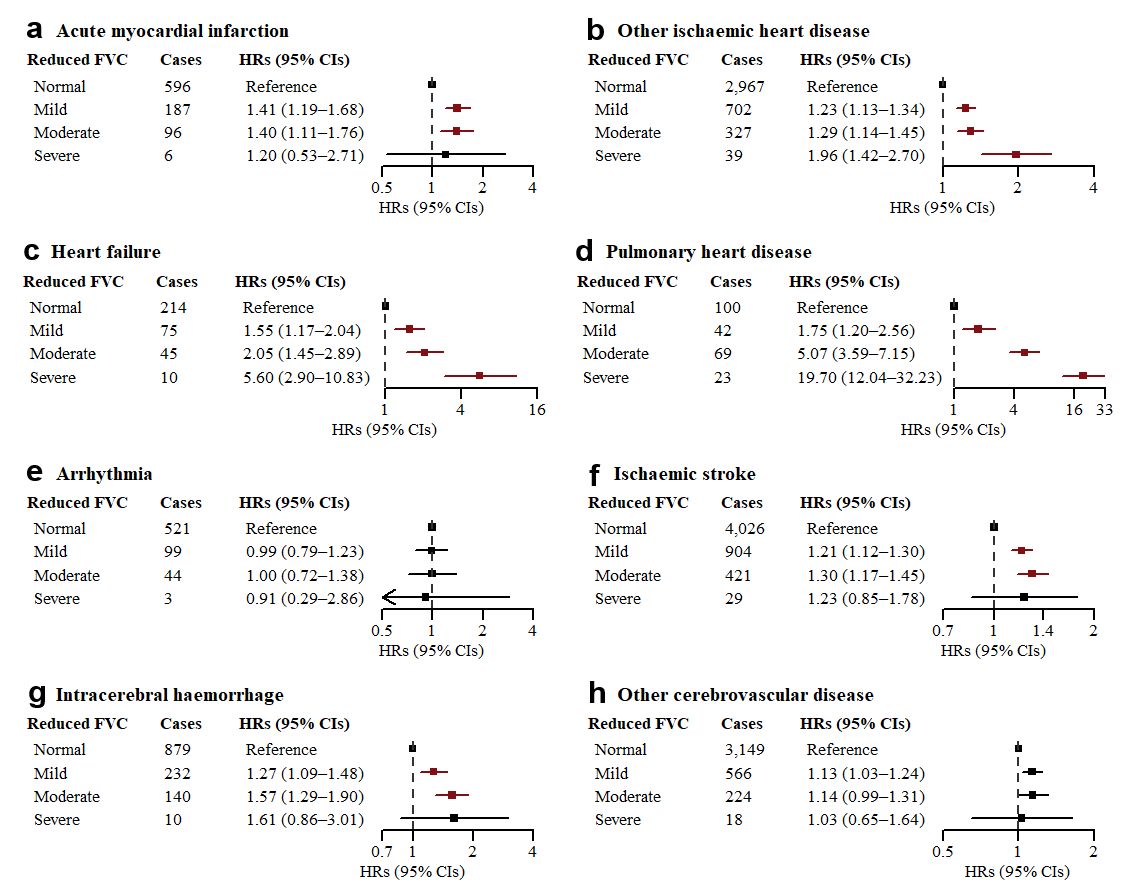


**Supplementary Fig. S5: Associations between severity of reduced FVC and CVD outcomes among males without AFO**

AFO=airflow obstruction; CI=confidence interval; CVD=cardiovascular disease; FEV1=forced expiratory volume in 1 second; FVC=forced vital capacity; HR=hazard ratio.

"Normal" refers to FEV1/FVC ≥ 0.7 and FVC z-score > –1.65; "Mild" refers to FEV1/FVC ≥ 0.7 and –2.5 ≤ FVC z-score ≤ –1.65; "Moderate" refers to FEV1/FVC ≥ 0.7 and –4 ≤ FVC z-score < –2.5; "Severe" refers to FEV1/FVC ≥ 0.7 and FVC z-score < –4.

The HRs (95% CIs) in red were statistically significant after Bonferroni correction (<0.0062). Multivariable models were adjusted for age, level of education, occupation, marital status, household income, alcohol consumption, physical activity, intake frequencies of red meat, fruits, and vegetables, body mass index, waist circumference, fuel types currently used in cooking, fuel types currently used in heating, years of cooking with solid fuels, years of heating with solid fuels, stove ventilation in the baseline house, passive smoking, and family histories of heart disease and stroke.


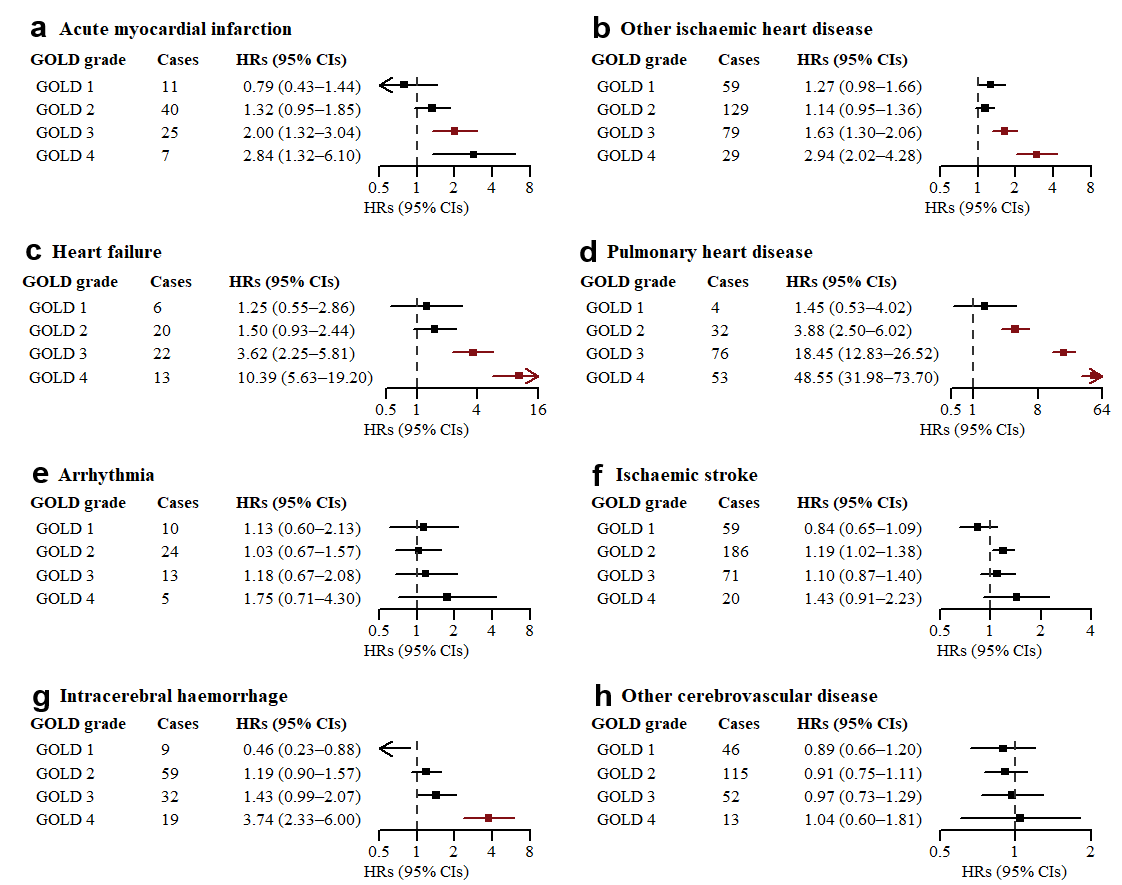


**Supplementary Fig. S6: Associations of GOLD grades of AFO and CVD outcomes among males** **without RSP**

AFO=airflow obstruction; CI=confidence interval; CVD=cardiovascular disease; FEV1=forced expiratory volume in 1 second; FEV1%P=forced expiratory volume in 1 second (FEV1) percent predicted; FVC=forced vital capacity; GOLD=Global Initiative for Chronic Obstructive Lung Disease; HR=hazard ratio; RSP=restrictive spirometric pattern.

"GOLD 1" refers to FEV1/FVC < 0.7 and FEV1%P ≥ 80%; "GOLD 2" refers to FEV1/FVC < 0.7 and 50% ≤ FEV1%P < 80%; "GOLD 3" refers to FEV1/FVC < 0.7 and 30% ≤ FEV1%P < 50%; "GOLD 4" refers to FEV1/FVC < 0.7 and FEV1%P < 30%.

The HRs (95% CIs) in red were statistically significant after Bonferroni correction (<0.0062). Multivariable models took those with FEV1/FVC ≥ 0.7 and FVC ≥ 80% predicted (the "normal" group) as the reference, and were adjusted for age, level of education, occupation, marital status, household income, alcohol consumption, physical activity, intake frequencies of red meat, fruits, and vegetables, body mass index, waist circumference, fuel types currently used in cooking, fuel types currently used in heating, years of cooking with solid fuels, years of heating with solid fuels, stove ventilation in the baseline house, passive smoking, and family histories of heart disease and stroke.

**
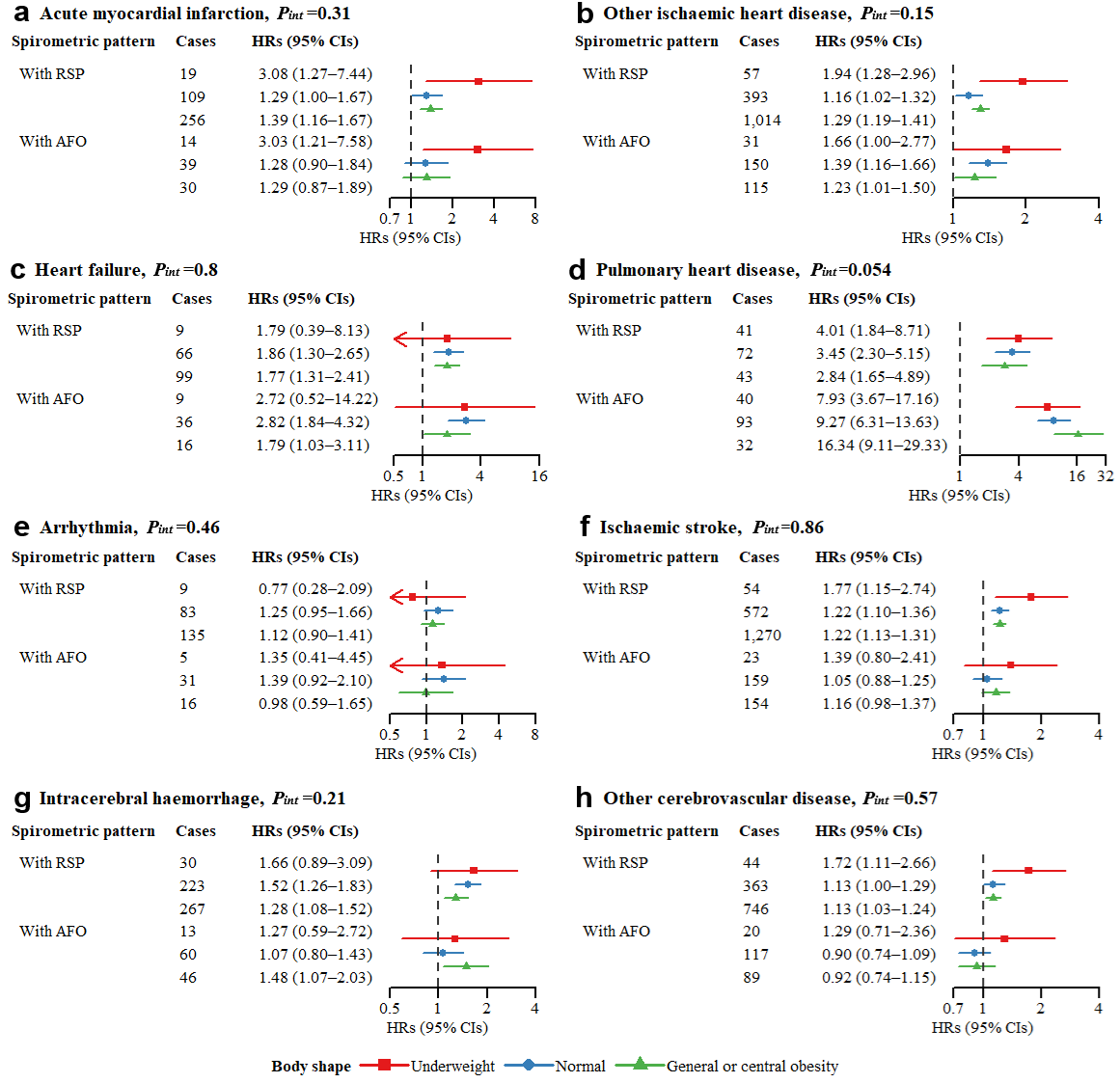
**

**Supplementary Fig. S7:** **Associations between spirometric pattern and CVD outcomes stratified by body shape among males**

AFO=airflow obstruction; BMI=body mass index; CI=confidence interval; CVD=cardiovascular disease; FEV1=forced expiratory volume in 1 second; FVC=forced vital capacity; HR=hazard ratio; RSP=restrictive spirometric pattern; WC=waist circumference.

In the spirometric pattern, "With RSP" refers to FEV1/FVC ≥ 0.7 and FVC < 80% predicted; "With AFO" refers to FEV1/FVC < 0.7.

In the body shape, "Underweight" refers to BMI<18.5 kg/m^2^; "Normal" refers to BMI 18.5–23.9 kg/m^2^ and WC<80/85cm; "General or central obesity" refers to BMI≥24 kg/m^2^, or BMI 18.5–23.9 kg/m^2^ and WC≥80/85cm.

Multivariable models took those with FEV1/FVC ≥ 0.7 and FVC ≥ 80% predicted (the "normal" group) as the reference, and were adjusted for age, level of education, occupation, marital status, household income, alcohol consumption, physical activity, intake frequencies of red meat, fruits, and vegetables, fuel types currently used in cooking, fuel types currently used in heating, years of cooking with solid fuels, years of heating with solid fuels, stove ventilation in the baseline house, passive smoking, and family histories of heart disease and stroke.
